# Benchmark of Density Functional Theory in the Prediction of ^13^C Chemical Shielding Anisotropies for Anisotropic Nuclear Magnetic Resonance-Based Structural Elucidation

**DOI:** 10.1021/acs.jctc.4c01407

**Published:** 2025-01-06

**Authors:** Anton Florian Ketzel, Xiaolu Li, Martin Kaupp, Han Sun, Caspar Jonas Schattenberg

**Affiliations:** † Institut für Chemie, Strukturelle Chemische Biologie und Cheminformatik, Technische Universität Berlin, Berlin 10623, Germany; ‡ Research Unit of Structural Chemistry & Computational Biophysics, Leibniz-Forschungsinstitut für Molekulare Pharmakologie, Berlin 13125, Germany; § Institute of Medical Sciences, The Second Hospital of Shandong University, 250033 Jinan, China; ⊥ Institut für Chemie, Theoretische Chemie/Quantenchemie, Technische Universität Berlin, Berlin 10623, Germany

## Abstract

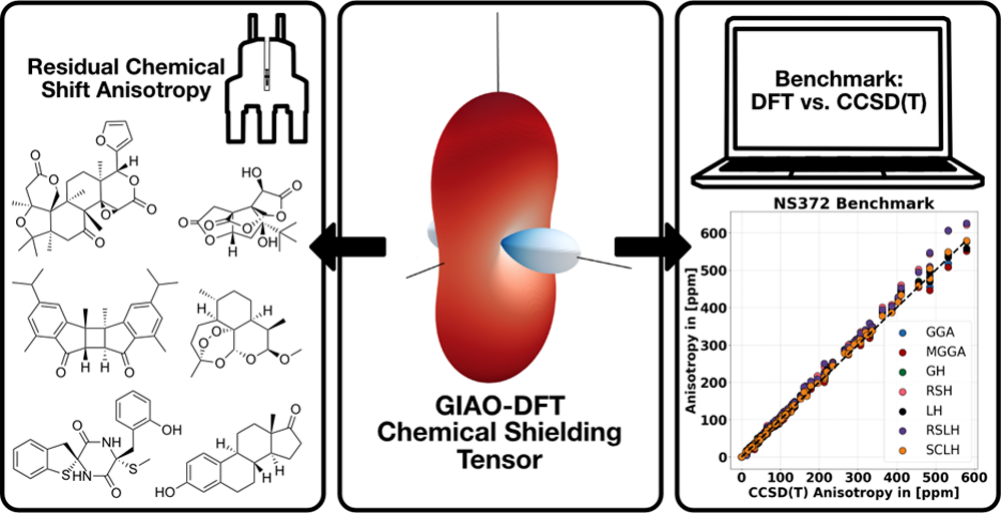

Density functional theory (DFT) calculations have emerged as a powerful theoretical toolbox for interpreting and analyzing the experimental nuclear magnetic resonance (NMR) spectra of chemical compounds. While DFT has been extensively used and benchmarked for isotropic NMR observables, the evaluation of the full chemical shielding tensor, which is necessary for interpreting residual chemical shift anisotropy (RCSA), has received much less attention, despite its recent applications in the structural elucidation of organic molecules. In this study, we present a comprehensive benchmark of carbon shielding anisotropies based on coupled cluster reference tensors taken from the NS372 benchmark data set. Additionally, we investigate the representation of the DFT-predicted shielding tensors, such as the eigenvalues and eigenvectors. Moreover, we evaluated how various DFT methods influence the discrimination of possible relative configurations using recently published ΔΔRCSA data for a set of structurally diverse natural products. Our findings demonstrate that accurate interpretation of RCSAs for configurational and conformational analysis is possible with semilocal DFT methods, which also reduce computational demands compared to hybrid functionals such as the commonly used B3LYP.

## 1 Introduction

Anisotropic nuclear magnetic resonance (NMR) spectroscopy in solution has been proven for over three decades to provide invaluable structural and dynamic information about biomolecules, particularly proteins and nucleic acids.^1−4^ However, more recently, the application of anisotropic NMR for small organic molecules has gained increasing attention, especially for its ability to determine the relative configuration of challenging organic molecules.^5−10^ This is because anisotropic NMR offers more long-range structural information compared to isotropic NMR parameters, such as chemical shift, *J*-coupling, and distances from the nuclear Overhauser effect.^11,12^ Moreover, beyond the determination of relative configurations, anisotropic NMR enables the accurate characterization of the three-dimensional conformational structure of the molecules.^13−15^

In isotropic media, under which most standard solution NMR experiments are conducted, anisotropic interactions are typically averaged out due to free molecular tumbling.^16^ However, when alignment of the sample is introduced, for example, as in solid-state NMR, or if alignment media are introduced in solution,^4,17^ shielding anisotropy becomes directly observable and manifests itself in three interactions: (i) dipolar coupling (DC), (ii) chemical shift anisotropy (CSA), and (iii) for nuclei spins  quadrupolar coupling (QC).^16,18,19^ Because these interactions can lead to excessive line broadening and thereby decrease the sensitivity dramatically, a lot of effort has been made in the field of solid-state NMR to suppress these interactions, e.g., by magic angle spinning.^20^ In anisotropic solution NMR, where for small molecules, the anisotropy is introduced by the alignment medium,^21^ these media are specifically designed to provide relatively weak alignment.^22^ As a result, these interactions, largely reduced in size, can subsequently be measured as residual dipolar coupling (RDC), residual chemical shift anisotropy (RCSA), and residual quadrupolar coupling.^19^

As interpreting anisotropic NMR data, such as RDCs, requires determining an alignment tensor, which is a three-by-three matrix, a sufficient number of nonlinear RDC data sets is necessary.^23,24^ This can be problematic for molecules composed predominantly of quaternary carbons.^5,6^ In these cases, RCSAs offer valuable complementary structural information that can be directly derived from one-dimensional ^13^C spectra. Recently, despite the low natural abundance of ^2^H, protocols using ^2^H-RQC for the stereochemical elucidation of natural products have emerged, which, however, have not seen broad adoption at this point.^25^ In contrast to RDCs, where the data can be directly employed in the structural elucidation process, RCSAs require the full chemical shielding tensor (CST) for their interpretation. Alternatively, the CSTs of organic molecules can be derived from RCSAs if the orientation of the analytes is known.^26^

Furthermore, the analysis of RCSAs is, contrary to the chemical shielding constant, dependent on the full tensorial properties of the CST. Therefore, accurate CST values are necessary for interpreting experimental RCSAs.^23^ Initially, in the analysis of protein RCSAs, CSTs obtained from solid-state NMR were used and applied to analogous molecular functional groups.^27^ This approach is not feasible for small organic molecules; therefore, the analysis relies on CSTs from computational methods.^5^ Highly accurate computational methods, such as coupled cluster theory, particularly in its CCSD(T) formulation, are renowned for achieving chemical accuracy.^28−30^ However, their application in the calculation of chemical shielding tensors of even moderately sized organic molecules is prohibitively expensive due to their significant computational demands. This becomes even more problematic as RCSA analysis potentially requires the inclusion of a larger number of molecular configurations.^7,10,31−33^ For this reason, usually more cost-efficient approaches have to be employed. The most commonly used method to date is density functional theory (DFT), specifically in its reformulation by Kohn and Sham (KS), due to its favorable cost/performance ratio, which has also been used extensively in the field of RCSAs.^5−10,31−36^ In KS-DFT, the largest part of the electronic energy is calculated exactly, and only nonclassical contributions (exchange, correlation, and a small part of the kinetic energy) are gathered in an approximate term, the exchange–correlation functional or density functional approximation (DFA).^37^ However, one major challenge of KS-DFT (and inherently the DFAs) is that no clear-cut recipe for a systematic improvement of functionals with guaranteed performance increase exists, which makes benchmarking of density functionals essentially unavoidable.

This necessity is reflected, for example, in many benchmark studies focusing on isotropic shielding constants;^38−48^ however, less attention has been paid to tensor properties such as chemical shielding anisotropies (CSAs) and RCSAs: (I) A limited number of studies focusing on CSAs have been reported. These studies are limited to a smaller set of DFAs benchmarked against experimental solid-state NMR chemical shielding tensors, which might introduce environmental effects and may mask the true performance of different functionals due to error compensation.^49−54^ (II) In the prediction of RCSAs, DFAs have commonly been chosen on the basis of their performance in predicting isotropic shielding constants.^13,36,55^ Presumably because overall few studies investigate the performance of different density functionals specifically for the analysis of RCSA and, moreover, these studies only evaluated a limited number of DFAs (mostly global hybrid functionals) and focused only on limited examples or single molecules.^7,8^ Nevertheless, successful applications of B3LYP and mPW1PW in the prediction of RCSAs have been reported.^6−9,32−36^ However, a systematic benchmark might enable us to find a DFA that (i) produces results closer to the experimentally measured RCSA or (ii) achieves similar accuracy with lower computational cost, especially since recent studies show that such global hybrid constructions perform only mediocrally. Yet modern DFAs, such as local hybrid (LH) functionals (especially in their strong correlation generalization) or mGGAs employing current-density response via their kinetic-energy density contribution, are among the most accurate DFAs up to rung 4 on Perdew’s ordering scheme for the calculation of isotropic shielding, but also of other magnetic resonance properties.^46,56,57^

To fill the gap in these anisotropic NMR properties, in this study, we (i) present a systematic benchmark of various DFAs for the calculation of carbon shielding anisotropies and their description of shielding tensor properties, in comparison to high-level CCSD(T) benchmark data and (ii) investigate the influence of these tensor properties, and their description by different DFAs in the stereochemical elucidation of a series of structurally diverse natural products.

## 2 Theory

### 2.1 Tensorial Properties of the CST

Based on perturbation theoretical considerations, the NMR shielding tensor of nucleus *K* within its Cartesian coordinate frame (*i*, *j* ∈ {*x*, *y*, *z*}) can be expressed as the second derivative of the energy with respect to the external magnetic field **B** and nuclear magnetic moment **M**_*K*_
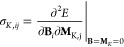
1The shielding tensor can be separated into symmetric and antisymmetric contributions
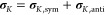
2where
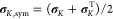
3
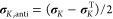
4

**Figure 1:**
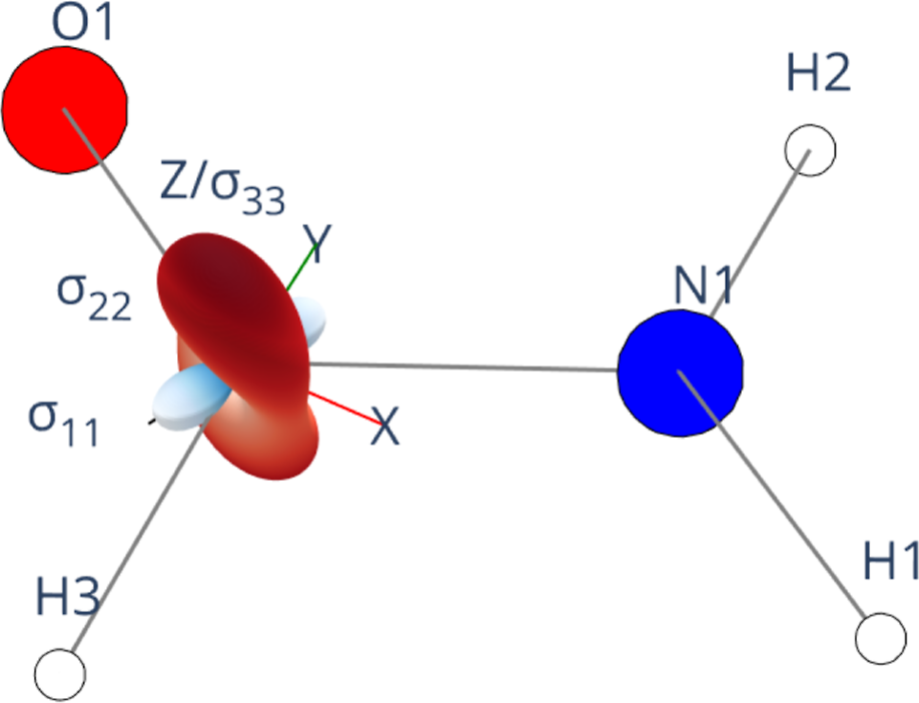
CCSD(T) CST for the carbon atom of HCONH_2_ visualized according to a protocol by Zurek et al.^58^ using Python. The positive values of the CST in polar coordinates are shown in red and the negative values in blue. Furthermore, the eigenvalues as well as the Cartesian axis system are shown.

Diagonalization of the shielding tensor gives its principal components (PCs) (σ_*II*_, *I* ∈ {*X*, *Y*, *Z*}, where *X*, *Y*, and *Z* are the Cartesian components of the PC frame), associated with eigenvectors describing the directions of the PCs within the molecular frame, as exemplarily visualized for the carbon shielding tensor in HCONH_2_ in Figure 1.

Throughout this work, the PCs are obtained as the eigenvalues of the symmetric contribution, **σ**_*K*,sym_.

The anisotropy of the shielding tensor in terms of the PCs can be defined as
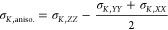
5where the Haeberlen convention for the ordering of the PCs can be employed, given by
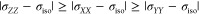
6This definition, however, allows for either positive (σ_*ZZ*_ > σ_iso_) or negative (σ_*ZZ*_ < σ_iso_) anisotropies. In cases where two PCs of opposite sign are almost equidistant to σ_iso_, the situation may arise that the change of sign becomes method or functional dependent, which can lead to strongly deviating results with almost identical PCs (see also Section S2 of the Supporting Information for a more detailed description).

For this reason, we use an alternative definition (sometimes called the Maryland convention, where usually numbers are used instead of letters to denote the eigenvalues),^59^ which also employs eq 6, but chooses σ_33_ as the largest value of the PCs, as
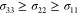
7Definitions with a reversed ordering have been proposed, but have not been further explored within this work.^60^ Additionally, we employ the definition of the tensor anisotropy used in Turbomole.(61,62)
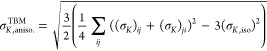
8

### 2.2 Residual Chemical Shift Anisotropies

The chemical shift of a nucleus measured under isotropic conditions and again after creating an anisotropic environment, for example, by adding a weakly aligning medium, exhibits a characteristic shift change given by
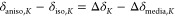
9where δ is the chemical shift for nucleus *K* and Δδ_media,*K*_ is the isotropic chemical shift change introduced by the chemical environment of the medium, which can be reduced by appropriate referencing schemes,^63^ and Δδ_*K*_ the chemical shift change due to the anisotropic interaction.^5^ Δδ_*K*_ depends on the orientation of the molecules, which can be described by the Saupe order matrix **S** with elements
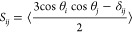
10where θ describes the angle between the molecular axis and the magnetic field, and δ_*ij*_ is the Kronecker delta function.^18^ For a more intuitive description, the Saupe order matrix is often converted into the alignment tensor, by .

With the Saupe order matrix known, one can calculate the chemical shift changes directly from the chemical shielding tensors using the Frobenius inner product
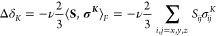
11in which ν is the Larmor frequency of the nucleus. In practice, however, the Saupe order matrix cannot be determined a priori and must be approximated. This is often done by minimizing the difference between the experimentally measured RCSA and the back-calculated RCSA from the CST. For a rigid molecule with negligible internal motion, the Saupe order matrix is identical for all nuclei, allowing for the setup of a system of linear equations with the same Saupe matrix for all nuclei. Additionally, by taking the properties of the Saupe order matrix into account, only 5 of the 9 elements of the matrix are linearly independent.

Therefore, for a molecule with *C*_1_ symmetry, at least five independent RCSAs are required to solve the system by calculating the inverse of the matrix constructed from the CST elements. In practice, however, more than five RCSAs are typically used for the determination of the tensor. To find a solution to this overdetermined system in a least-squares sense, singular value decomposition (SVD) can be employed to compute the Moore–Penrose pseudoinverse^64−66^ for the calculation of the Saupe order matrix.^23^

#### 2.2.1 Experimental Considerations

Because the introduction of an alignment medium introduces the isotropic chemical shift change Δδ_media,*K*_ in the analyte, which is not due to the anisotropic interaction, it was found to be beneficial to use ΔRCSA, which are calculated by an internal referencing of the RCSA to a carbon nucleus in the analyte.^63^ Similarly, instead of comparing the isotropic chemical shift to the anisotropic chemical shift, it was found to be beneficial to use the chemical shift change under two different alignment conditions,^5^ as reflected in
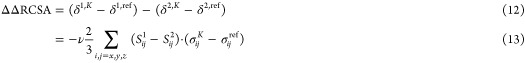
where δ^1,*K*^ and δ^1,ref^ are the chemical shifts of nucleus *K* and of the reference carbon nucleus under the stronger alignment condition 1, and δ^2,*K*^ and δ^2,ref^ are the chemical shifts under the weaker alignment condition 2.

In most cases, the difference between the experimental RCSA and back-calculated RCSA is quantified in terms of a normalized root-mean-square-error (RMSE), the so-called quality (*Q*)-factor^27^
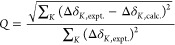
14

### 2.3 Remarks on DFT

As stated in the introduction, this study has the objective of benchmarking density functionals or DFAs. These are the approximative term in KS DFT, describing the nonclassical contributions to the electronic energy. Such DFAs can be classified by hierarchical ordering for example on the basis of their ingredients (density, first and second derivatives of the density, or orbital dependence) by schemes such as Perdew’s famous ladder approach,^67^ or by more elaborate means relying on their orbital dependence and the admixing of exact-exchange contributions to the functional form.^68^ We will not discuss all employed functionals here in detail, however, we refer the interested reader to recent reviews covering the theoretical basis,^69^ and a broad overview of many DFAs,^70^ as well as perspectives of density functional development,^71^ which give an overview of (semi)local (LDA, GGA, and mGGA functional), hybrid functionals, and range separated (RS) hybrid functionals. For more details on the employed LH functionals and their range-separation and strong-correlation generalizations (SC), we refer to two relatively recent reviews, which cover the general idea, development, implementation, and application of LHs,^72^ as well as an overview of recent RS and SC developments.^73^

## 3 Computational Details

### 3.1 Computational Settings

The first part of the article (Section 4.1) comprises a benchmarking of various DFAs against the previously computed 93 shielding data for the ^13^C subset of the NS372 shielding and shift test set of CCSD(T) quality.^46^ The original data were computed using CFOUR version 1.2,^118^ as described in detail in the original publication. To keep the data concise, we focus on carbon shielding data, as this nucleus is most prominently employed in the calculation of RCSAs in the configurational determination of natural products.^5,6,8−10,31^ To ensure direct comparability and avoid the necessity of shielding tensor rotations, we employ the same structures but partly different orientations as the original NS372 data, now matching the orientations of the CCSD(T) tensors.

Throughout, DFA calculations have been performed with Turbomole, version 7.4 or newer,^61,119^ using the same computational setting as in the isotropic benchmark,^46^ i.e., Turbomole grid sizes “3”,^120^ convergence criteria of the SCF energy of 10^–9^ and density matrix differences of 10^–7^ were used for all calculations. The resolution of the identity for the Coulomb contribution has been used.^121^ Throughout the benchmark, pcSseg-3 basis sets in connection with “universal” auxiliary basis sets have been employed, which were shown to be close to basis set completeness for the studied set of molecules.^122,123^ To save computational resources, throughout Section 4.2 the pcSseg-2/“universal” basis set combination is employed, as the influence of the basis-set on the predicted *Q*-factors in the configurational assignment is negligible (see Table S3 in Supporting Information). Gauge-including atomic orbitals have been used throughout the coupled-perturbed equations to obtain gauge independent results.^124−126^ The convergence of the SCF and shielding computations was checked carefully for all calculations.

To capture potential solvent effects of the experimental setup of the natural products, the implicit conductor-like screening model (COSMO) with a dielectric constant of ϵ = 32.613 has been employed for the chemical shielding calculation, in accordance with prior studies on RCSA.^6,9^

The employed methods and density functionals of the benchmark study are listed in Table 1. To the best of our knowledge, to date, no detailed analysis and benchmark of shielding anisotropies and tensor properties has been published; for this reason, we include a wide set of methods and DFAs, comprising also Hartree–Fock, LDA, and GGAs, which were less successful in isotropic benchmark studies for shieldings and shifts.^46,127,128^ However, as the methods should be applied to natural products in the discrimination of configurations, we do not include more expensive virtual-orbital dependent methods such as MP2 or double-hybrid functionals and restrict ourselves to the first four rungs on Perdew’s “Jacob’s ladder” hierarchical ordering.^67^ We additionally include the GGA-based hybrid functional mPW1PW91, which is an often employed DFA in the calculation of RCSAs, and regularly compared to B3LYP.^13^

**Table 1:** Employed Density Functionals [Including Hartree–Fock (HF)], Divided into Strong Correlation Local Hybrid Functionals (scLH), Range Seperated Local Hybrid (RSLH), Local Hybrid Functionals (LH), Range Seperated Hybrid Functionals (RSH), Global Hybrid Functionals (GH), and Semilocal Functionals (mGGA, GGA, and LDA)^a^

functional	type	references
scLH23t-mBR	scLH	(74)
scLH22t	scLH	(75)
scLH22ta	scLH	(75)
scLH21ct-SVWN-m^b^	scLH	(76)
ωLH22t	RSLH	(77)
LH20t	LH	(78)
LH14t-calPBE^b^	LH	(79)
LH12ct-SsirPW92^b^	LH	(80)
LH12ct-SsifPW92^b^	LH	(80)
LH07t-SVWN^b^	LH	(81,82)
LH07s-SVWN	LH	(83)
mPSTS-noa2^c^	LH	(84,85)
LHJ14	LH	(86)
ωB97M-V	RSH, mGGA	(87)
CAM-B3LYP	RSH, GGA	(137)
TPSSh	GH, mGGA	(88,89)
PW6B95	GH, mGGA	(90)
M06-2X	GH, mGGA	(91)
M06	GH, mGGA	(91)
MN15	GH, mGGA	(92)
B3LYP^d^	GH, GGA	(93,94)
PBE0	GH, GGA	(95)
mPW1PW91	GH, GGA	(96)
B97-2^e^	GH, GGA	(97)
BHandHLYP	GH, GGA	(98)
TPSS	mGGA	(88,89)
B97M-V^e^	mGGA	(99)
VSXC^e^	mGGA	(100)
MN15-L	mGGA	(101)
M06-L	mGGA	(102)
τHCTH^e^	mGGA	(103)
rSCAN^e^	mGGA	(104)
HCTH^e^	GGA	(105,106)
B97-D^e^	GGA	(107)
BP86	GGA	(108–110)
PBE	GGA	(111)
BLYP	GGA	(108,112)
KT1^d^	GGA	(113)
KT2^d^	GGA	(113)
KT3^d^	GGA	(114)
SVWN	LDA	(115,116)
Hartree–Fock		

a For details, see the respective references.

b Functionals also abbreviated as scLH21ct, LH12sir, LH12sif, LH14t, and LH07t.

c See ref (85) for further details of the noa2 model of mPSTS.

d B3LYP version of Turbomole using VWN5, as discussed in Hertwig and Koch.^94^

e Used via LibXC.^117^

All RCSAs have been analyzed using the SVD procedure, as implemented in the ConArch^+^ software package.^129^ Furthermore, the single alignment tensor approximation for compounds where a conformational ensemble was necessary was used throughout. Error analysis was automatically done by ConArch^+^ using an estimated experimental error of 0.5 Hz in a Monte Carlo approach. *Q*-factors are reported as mean *Q*-factors from the Monte Carlo procedure. To ensure consistency with the already published data, the SVD fitting of the RDC data from the publications^6,9^ has been repeated and the cosine similarities between the RDC- and RCSA-derived alignment tensors have been calculated for all natural products and DFAs (see Table S3 in the Supporting Information).

Throughout, the density dependence of the VV10 dispersion model is not reflected in the shielding computations and does contribute only indirectly via the SCF orbitals. Furthermore, the Dobson model for gauge independence of the kinetic energy density (τ) is used, which introduces current-density response.^130−132^ We nevertheless skip the prefix “c” as employed in previous studies using the same gauge correction for the kinetic energy density,^85,133,133−135^ as we do not employ comparisons using the Maximoff–Scuseria model for τ.^136^

### 3.2 Set of Experimental ΔΔRCSA

To study the effect of DFAs on the analysis of RCSA, a small but structurally diverse set of natural products was selected from our earlier described analyses of RCSAs. For this purpose, the published experimental ΔΔRCSA data and molecular structures have been used for β-artemether **1**, bilobalide **2**, estrone **3**, limonin **4**, spiroepicoccin A **5**, and weizhouochrone A **6**.^6,9^ The ΔΔRCSA data of these six compounds were all measured in a liquid crystal-based medium formed by AAKLVFF^17^ in methanol.

Furthermore, to investigate the potential of DFAs in discriminating configurations by their *Q*-factor, we looked at one alternative configuration for each natural product. For molecules **2** and **6**, where multiple possible alternative configurations were discussed in previous publications,^6,9^ only the alternative configuration with the second-lowest *Q*-factor was chosen for the present investigation.

All chemical structures are listed in Figure S4 in the Supporting Information, and in the following, (**a**) denotes the correct configuration and (**b**) the alternative configuration. The reference carbon atoms for the ΔΔRCSA data have been chosen based on the highest performance in their respective publications.

## 4 Results

### 4.1 Benchmark Results for the ^13^C Subset of NS372

#### 4.1.1 Chemical Shift Anisotropy

**Figure 2:**
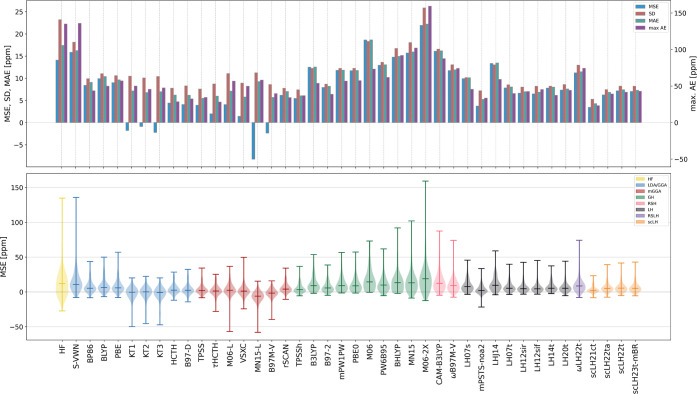
(Top) Statistical data, (bottom) violin plots of the mean signed errors with respect to CCSD(T) reference data of positive definite shielding anisotropies according to eqs 5 and 7.

To gain first insights into the performance of the set of DFAs for tensor anisotropies, we compare deviations of the DFA data from the CCSD(T) reference data of the ^13^C subset of NS372 based on the Maryland convention, as expressed in eq 7. We report the discussions and data for the Haeberlen convention and Turbomole definitions (eqs 6 and 8) in Supporting Information, as the anisotropies provided by the Haeberlen convention are hampered by the inherent problem of sign changes (see Section 2) and they therefore provide a poor representation of the actual tensor descriptions, while the Turbomole defined anisotropies provide data which is comparable to the data obtained using the Maryland convention and negligible additional insight is gained (see Section S2 and Figures S1 and S2 therein). Statistical data [mean signed error (MSE), mean absolute error (MAE), standard deviation (SD), and maximal absolute error (max. AE)], together with violin plots of the MSE, are presented in Figure 2; the full statistical data alongside a two part table highlighting the 10 DFAs with lowest MAE and max. AE are gathered in Tables S1 and S2, respectively, in Section S1 of the Supporting Information. Additional cross correlation plots of the statistical data are provided in Figure S5 in Section S6 of the Supporting Information.

Starting the discussion by analyzing first at the MAEs combined with the SDs, we find scLH21ct-SVWN-m to show the lowest MAE and SD (as well as the lowest max. AE), outperforming the following best functionals in these metrics by 0.98 and 1.92 ppm (as well as 10.23 ppm), respectively. The mPSTS-noa functional provides the second lowest MAE and SD. Notably, it provides an MAE below 5.3 ppm and a SD below 7.2 ppm, and it also occupies a high rank (rank 5) in the max. AE data (see also below). Seven additional functionals give MAEs within 2 ppm of scLH21ct-SVWN-m, i.e., below 6.3 ppm: these are (a) TPSS and TPSSh, which follow their LH descendant mPSTS-noa2 closely in MAE (ranks 3 and 7) and SD (ranks 5 and 3), respectively. (b) Three additional mGGA models B97M-V, VSXC, and τHCTH. Of these, the first two also occupy top ranks in the overall isotropic shielding comparison and show a relatively good performance in the isotropic shielding data of the carbon nucleus as well (generally only upon proper inclusion of current-density response).^46^ However, they occupy middle ranks in SD and give max. AE close to 40 and 50 ppm, respectively. In comparison to these two functionals, τHCTH is better in max. AE.

(c) Two GGAs B97-D and HCTH, of which the latter also gives a SD in the range of the best performing functionals for the metric. Notably, these two models also give the third and fourth lowest max. AE of all tested functionals, even slightly outperforming mPSTS-noa2 (see below).

Not far behind (MAE roughly within 3 ppm in comparison to scLH21ct-SVWN-m) are top performing LHs of the overall isotropic shielding data, LH12ct-SsirPW92 and LH12ct-SsifPW92 (which also rank 1 and 2 in the isotropic carbon shielding data of the DFAs discussed here),^46^ scLHs, mGGAs rSCAN, and M06-L, as well as specialized GGAs of the KT series. These functionals all show MAEs below 7.4 ppm, while the remaining t-LMF-based LH and functionals give MAEs below 8.1 ppm.

Notably, rSCAN, the t-LMF-based LHs and scLHs give comparably low SD, still occupying ranks in the upper quartile and half of the SD comparison. However, M06-L and KT functionals are worse here and occupy ranks in the lower half. Interestingly, both scLHs of the scLH22 series show a slight advantage over their parent LH20t, which can be attributed mostly to slightly different descriptions in carbenes, carbenium ions, carbanions, and carbons directly bound to hetero atoms, such as oxygen, sulfur, or chlorine. The effects are mostly subtle but clearly visible in the statistical data. As expected, the effect is more pronounced for scLH22ta which, in contrast to scLH22t, uses an undamped strong-correlation correction and for which larger corrections have also previously been reported for isotropic shielding data and magnetizabilities.^56,57^ Similarly, the data for the second damped scLH scLH23t-mBR follow the trends of scLH22t, and they overall perform very similarly, separated by only one rank in the SD and max. AE, and occupying neighboring ranks in the MAE.

Further GGA models and the mGGA MN15-L range between 9 and 11 ppm in MAE, while GHs, with the exception of B97-2, exceed 10 ppm. Also range-separated hybrids as well as LHJ14 and SVWN fall into an error range of 11–17 ppm, while Hartree–Fock and M06 exceed 17 ppm. M06-2X shows the highest MAE, above 22 ppm (combined also with the largest SD and max. AE). Overall, DFAs of the lowest quartile (the 10 DFAs with the largest MAE) gave values above 12 ppm. SDs are still close to 10 ppm for the GGA models of the lower half and thus comparable to the KT series of functionals, but they become larger for SVWN, highly parametrized functionals of the Minnesota series, and also for GHs with increasing exact-exchange admixture: for PW6B95 (28%), the SD already exceeds 13 ppm, but for GHs with EXX above 40%, M06, and the RSH CAM-B3LYP (as well as for Hartree–Fock), SDs become larger than 15 ppm and increase up to 25.9 ppm for M06-2X.

Turning now to the MSE, also reflected in the violin plots in the bottom panel of Figure 2, we do not find a systematic underestimation for all DFAs, as observed for the isotropic data. Instead, for B97M-V, MN15-L, and the KT functionals, negative MSEs are observed [i.e., CCSD(T) anisotropies are underestimated], where MN15-L shows the overall most systematically negative MSE not far from its MAE value and the KT functionals and B97M-V have MAEs closer to zero, indicating that their distribution is closely scattered around the reference data. Also, DFAs such as VSXC and τHCTH also exhibit MSEs that are close to zero but positive (so a slight overestimation of the anisotropies is observed). For most other DFAs, the MSEs are larger, indicating overestimation of the anisotropies, and except for some of the better performing DFAs, such as TPSS, B97-D, HCTH, rSCAN, and M06-L, the deviation between the absolute MSE and MAE is small, indicating a tendency toward systematic overestimation of the shielding anisotropies.

Maximum absolute errors of all methods ranged from 23.2 to 159.2 ppm. As stated above, scLH21ct-SVWN-m occupies the top rank with a max. AE of 23.2 ppm, followed by the mGGA τHCTH and the GGAs HCTH and B97-D on ranks 2, 3 and 4, already with max. AEs slightly below and above 30 ppm. The LH mPSTS-noa2 as well as the mGGAs rSCAN and TPSS follow up to an error range of 35 ppm. The mGGA B97M-V, GHs TPSSh, and B97-2, the t-LMF-based LHs with lower exact-exchange admixture (LH07t and LH14t) and the undamped scLH22ta all remain below 40 ppm in max. AE. DFAs such as the t-LMF-based LHs with larger exact-exchange admixture, the damped scLH22t, most GGAs, and VSXC all exhibit max. AEs above 40 ppm and up to 50 ppm, whereas the remaining GHs, mGGAs, and RSHs all exceed this error mark, sometimes significantly so. Notably, of the tested functionals and methods, four surpass a max. AE of 100 ppm: SVWN, HF, and two Minnesota functionals with a large exact-exchange admixture.

#### 4.1.2 Cosine Similarities of Eigenvectors (Direction Criterion) and Shielding Tensors (Shape Criterion)

##### 4.1.2.1 Direction Criterion

Looking still at the ^13^C subset of NS372, we include the cosine similarities of the PC vector as a measure of the tensor shape and cosine similarities of the eigenvectors as a criterion of tensor orientation. Of the resulting cosine similarities for the eigenvector products, we exclude those for which the eigenvalues are close to degeneracy (eigenvalue differences < 3 ppm), as in these cases, the tensor PCs are not as clearly defined, i.e., they are potentially omnidirectional, without impacting the overall tensor shape. Due to local symmetry (i.e., symmetric tensors in the *XY* plane) of the *X* and *Y* eigenvectors, 63 and 62 of the 93 CCSD(T) ^13^C carbon data points, respectively, remain for the analysis employing the described exclusion criterion. The *Z* component is less affected, and here only 3 data points are excluded (CH_4_, CF_4_, and CHF_3_; the first two are spherically symmetric, and the third shows a *YZ* degeneracy of the eigenvalues in the Maryland convention). As signs of eigenvectors are arbitrary, we computed both alignments and chose the larger cosine similarity, i.e., the smaller difference in angle.

**Figure 3:**
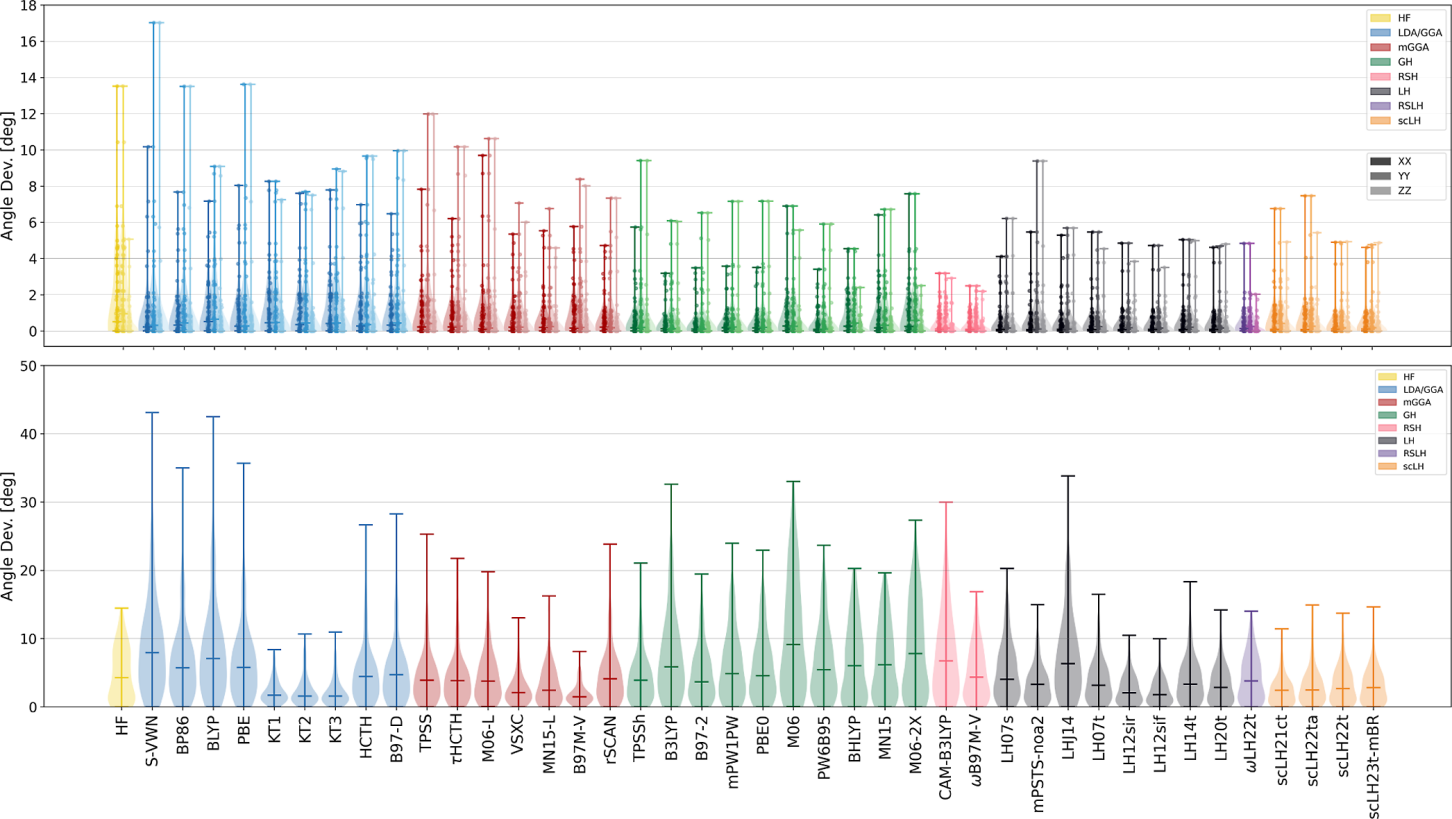
(Top) Violin plots of the cosine similarities of DFA and CCSD(T) tensor eigenvectors with the individual cosine similarities shown as a scatter plot on top; directions are shown in color shades. (Bottom) Violin plots of the cosine similarities of the full DFA and CCSD(T) tensors; mean absolute errors of the isotropic shielding data with respect to CCSD(T) reference data is shown as a scatter plot on top of the violin plots.

Starting the analysis of the cosine similarities of the PC eigenvectors, as shown on top of Figure 3, we note that the eigenvectors are comparably well reproduced with basically all methods and functionals, i.e., the PCs of the CCSD(T) reference and DFA tensors align very well. The density distribution of the violin plots centers close to the origin for all functionals, which is also reflected in the data points gathering predominantly below 4° [additional ordering the eigenvector cosine similarities into categories of increasing deviation from the CCSD(T) reference data is also shown in Table S4 in Supporting Information]. The largest deviations among all functionals and tensor directions are found for SVWN and amount to 17° in the *Y* eigenvector of the methyl groups in CMe_3_^+^ (excluding this cation reduces the maximum error of SVWN to roughly 10°). It should be emphasized that a *C*_*S*_ symmetric structure was employed in the NS372 benchmark set for this system instead of its true *C*_1_ symmetric ground state geometry to save computational resources, as the effects on the isotropic shielding data were observed to be negligible. It remains unclear whether the local symmetry would show better alignment for the true converged ground state structure. However, the alignment for other functionals seems to be unaffected, and the deviations are smaller.

Overall, the deviations in the cosine similarities of methods and functionals appear to decrease with the size of the eigenvalues: (a) the best agreement between CCSD(T) and computed eigenvectors is observed for the eigenvector describing the orientation of the largest eigenvalue σ_*K*,*ZZ*_. Of the 90 eigenvectors, between 81 and 88 cosine similarities of the eigenvectors are below 1°, which is reflected also in an average deviation of the cosine similarities below 1° for all tested methods and functionals for these eigenvectors (see Table S4 in the Supporting Information). (b) Cosine similarities for the *X*- and *Y*-components trail closely behind with average deviations exceeding 1° only in several (semi)local DFAs (however, VSXC, MN15-L, and rSCAN also provide average deviations below one degree in all three components). Hartree–Fock is the only method exhibiting an average deviation above 1.5° for its *X*- and *Y*-components (1.72 and 1.93°, respectively).

Comparing the individual deviations, the RSH functionals clearly show the best alignment of the eigenvectors of all tested methods and DFAs, with maximum deviations below 4° in all PC eigenvectors. Also, the t-LMF-based LH functionals and several GHs perform well, with only a few deviations exceeding the 4° mark. Particularly notable here are the LH12ct functionals, providing an accurate alignment below 4° in all *Z*-components, and similarly GHs with an exact exchange admixture between 20 and 25% which achieve a similar accuracy in the *X*-components. Deviations above 10° are generally rare and occur only sporadically with Hartree–Fock and some (semi)local functionals.

##### 4.1.2.2 Shape Criterion

Finally, we turn to the cosine similarities of the full unsymmetrized tensors, as shown at the bottom of Figure 3. We include those as a metric for the tensor shapes, i.e., deviations of the ellipsoids spanned by their eigenvectors from the reference tensors. Here, the full unsymmetrized tensors are employed throughout instead of the PC vectors, as both produce almost indistinguishable representations of the tensor shapes (see also the cross correlation of both metrics shown on the right-hand side in Figure S6 and Supporting Information).

Despite the fact that the shape criterion is a representation of the eigenvalues from which also the isotropic shielding data are calculated, both metrics are only loosely correlated when comparing their mean values calculated as the deviation from the CCSD(T) reference data. Indeed, the alignment of the mean data does not align linearly as expected for perfect correlation, but deviates in both relative directions (see left-hand side of Figure S6 in Supporting Information). These deviations from the diagonal appear to be a representation of different degrees of two limiting scenarios in the individual data: (i) for relative changes of the same magnitude for different sized PCs, deviations in σ_iso_ increase, while the cosine similarities are unaffected, and (ii) for symmetric shifts of the PCs canceling in the isotropic shielding result (i.e., the easiest case Δσ_33_ = −Δσ_11_), the deviations of the cosine similarities may increase significantly. These cases are, for example, relatively clearly reflected by the carbon shielding deviations and cosine similarities of C_2_F_4_ (Δσ_iso_ = 0.32 ppm, ΔΘ_T_ = 10.2°) and CF_2_ (Δσ_iso_ = 36.2 ppm, ΔΘ_T_ = 0.8°) for Hartree–Fock. However, a detailed analysis of these aspects of the shape and its representation by different DFAs is outside the scope of the present analysis, and we focus more closely on the functional overall performance.

In the following, we focus on the data with the largest relative shifts in the cross-correlation of the MAE of isotropic data and cosine-similarities: The overall similarity between both metrics appears to become more linear for the functionals, with larger deviations in the statistical data for tensor shapes and isotropic shieldings. However, deviations in the performance are visible for the better performing DFAs, for which the mean cosine similarities appear to increase slightly for functionals with some exact exchange admixture. HF stands out with the largest deviation between the two metrics, giving a relatively low MAE in isotropic shielding data but much larger deviations for the tensor shapes. The reason appears to be very large overall PCs, which cancel somewhat for isotropic shielding but are still reflected in the shape criterion. Similarly, for the best performing functionals, we find a slightly decreased performance with a larger exact-exchange admixture when comparing the LH12ct series of functionals and scLH21ct-SVWN-m to KT and B97M-V: where the latter two functionals are now on par or even better in the description of the shape metric. Due to the good reproduction of tensor shapes with semilocal DFAs, the KT and B97M-V functionals provide the overall lowest MCS_*T*_, closely followed by LHs and scLHs which, however, perform best for the isotopic shielding data.

##### 4.1.2.3 Concluding Remarks

Tapping the potential of DFAs for shielding anisotropies, it appears that overall semilocal constructions (except for some highly parametrized and KT functionals) perform better for anisotropic than for isotropic data and the deviation to the top performing models is hence smaller. This is also reflected in the cross correlation of the isotropic vs the anisotropic MAE (see Figure S7 in Supporting Information). Nevertheless, we find that the good performance for the isotropic shielding data and magnetizabilities of the undamped scLH model scLH21t-SVWN-m is preserved also for the anisotropic data. Besides, TPSS-based LH, GH, and semilocal functionals provide accurate results, whereas other functionals that are top performers for the isotropic data, such as the LH12ct series of LH functionals, B97M-V, and VSXC, still perform reasonably well but do not provide similarly outstanding performance as for the isotropic data. It should also be emphasized again that all calculations were checked carefully for convergence. Hence, outliers and larger error margins rather reflect a less accurate description of the electronic structure or at least larger deviations from the CCSD(T) reference data for the investigated shielding tensors by some of the benchmarked DFAs.

However, comparing further metrics related to the reproduction of tensor orientation and shape, we found the first to be reproduced very well with essentially all functionals, whereas the second is best reproduced with some of the best performing functionals from the anisotropic benchmark, but also with semilocal functionals such as B97M-V and KT.

### 4.2 Influence of DFT Functional on the RCSA-Based Conformational and Stereochemical Elucidation

**Figure 4:**
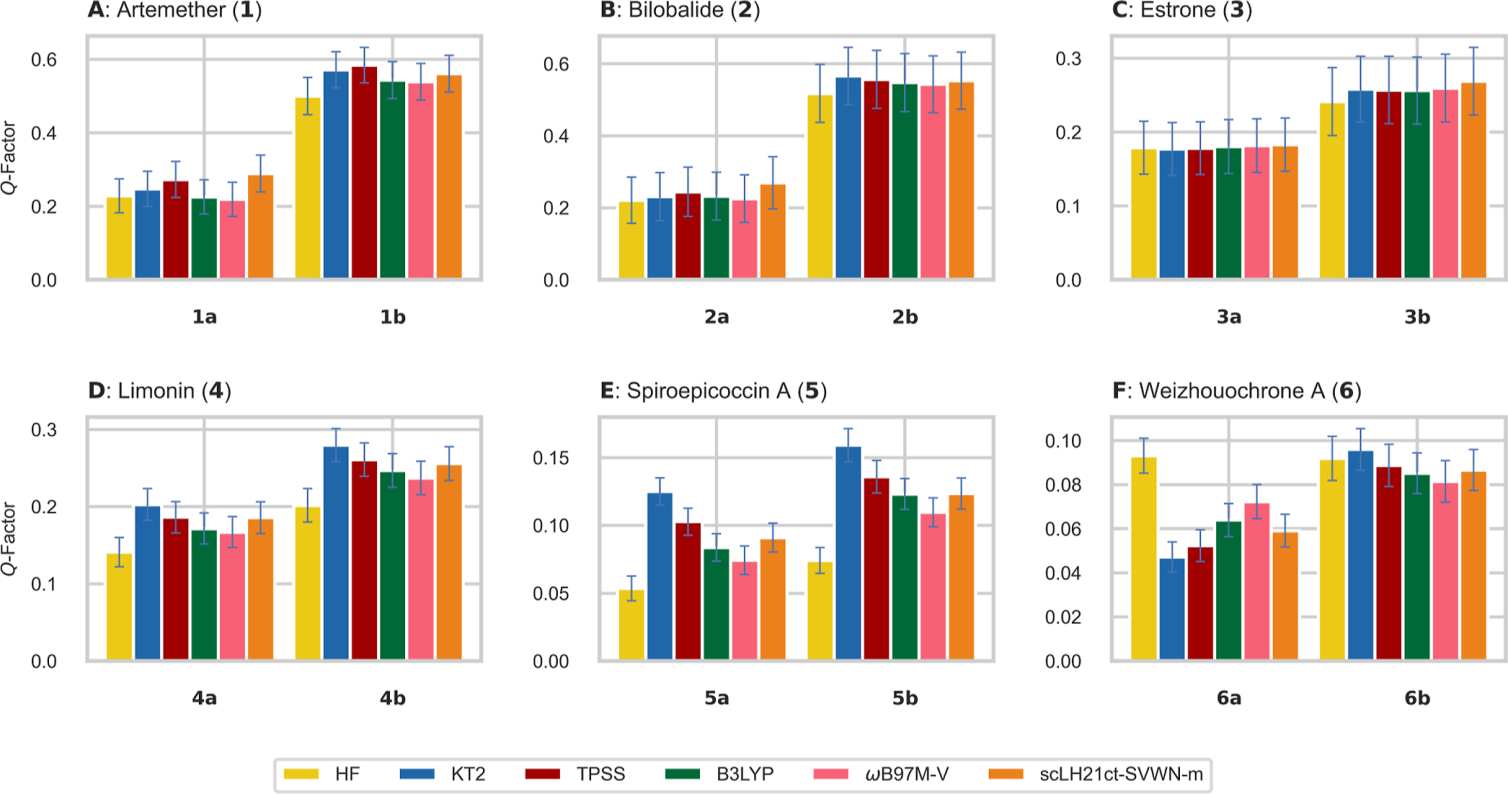
Comparison of *Q*-factors of six previously studied natural products **1–6** by ΔΔRCSA analysis. The *Q*-factors have been determined by an SVD approach using experimentally measured ΔΔRCSA,^6,9^ in combination with shielding tensors from Hartree–Fock, KT2, TPSS, B3LYP, ωB97M-V, and scLH21ct-SVWN-m. In all cases, the basis set pcSseg-2 has been used, and solvation effects have been treated with COSMO using parameters for methanol. The error of the experimentally determined ΔΔRCSA has been taken into account by a Monte Carlo sampling of the experimental value according to the estimated error.

As was mentioned in Section 1, the analysis of experimental RCSA necessitates accurate estimations of the CST. The comparison of the CSTs calculated with different DFAs to CCSD(T) reference data showed a more pronounced differentiation in the tensor shapes compared to the directions. For the latter, essentially all methods under study gave very similar results. Still, albeit with a small influence, a tensor with higher accuracy compared to the high-level CCSD(T) should, in principle, describe the experimentally measured RCSAs more accurately. In the following, the influence of the CST calculated with different DFAs will be investigated for a set of six structurally diverse natural products **1–6**. Here, we compare the *Q*-factors of the evaluated DFAs on the correct relative configuration (which has the lowest *Q*-factor among all possible configurations) along with another configuration that has the second lowest *Q*-factor; see Figure 4. It is important to note that all experimental RCSA data evaluated in this study were measured using a single alignment medium, AAKLVFF. This medium allows for the acquisition of highly accurate RCSA data without requiring any corrections during postacquisition data analysis. To extend this evaluation to other alignment media with varying degrees of order, further systematic studies are needed.

Starting the discussion with artemether (**1**) and bilobalide (**2**), an overall small influence of different DFAs on the configurational discrimination by the calculated *Q*-factors can be observed. Generally, the *Q*-factors for both configurations are smaller, when global or RS hybrid functionals (B3LYP, ωB97M-V) are employed. This does not affect the *Q*-factor difference; hence, no better discrimination is achieved. A similar trend, but more pronounced in terms of the absolute *Q*-factors, is observed for the natural products **4**, **5**, for which particularly B3LYP, ωB97M-V, and Hartree–Fock provide reduced *Q*-factors. However, this is similar for both configurations, yielding again no benefit to the discrimination. Also for estrone (**3**), the small differentiation of DFAs persists, only HF gives slightly worse and scLH21ct-SVWN-m slightly better discrimination in comparison with the other DFAs.

However, for weizhouochrone A (**6**), a distinct influence of the choice of DFA is visible. A clear distinction is achievable primarily with KT2 as well as the TPSS functional. While DFAs such as B3LYP and scLH21ct-SVWN-m also allow for discrimination, the degree of discrimination is noticeably less pronounced. Furthermore, although the correct configuration exhibits a lower *Q*-factor with ωB97M-V, the substantial overlap of the error margins precludes any definitive discrimination. Notably, the Hartree–Fock method entirely fails to distinguish between the configurations for **6**.

Finally, while the description of the individual *Q*-factors is only slightly affected by the chosen DFAs, essentially all functionals succeed in the discrimination of the tested natural products (see Figures S8–S13 in the Supporting Information). Only compound **6** was found to be more sensitive to the chosen DFA, for which both HF and RSH ωB97M-V provide inconclusive results. Intriguingly, DFAs employing exact exchange do not provide significantly enhanced accuracy. Hence, the additional computational effort of coupled perturbed Kohn–Sham equations, which are computationally demanding for exact-exchange dependent methods, but more efficient for current-dependent mGGAs such as TPSS, can be avoided entirely by choosing a GGA such as KT2. The decreased computational cost for the chemical shielding calculation is approximately 1:38 for KT2 compared to B3LYP and 1:12 for TPSS compared to B3LYP (see Table S6 in Supporting Information).

#### 4.2.1 Detailed Discussion of Weizhouochrone A

Weizhouochrone A stands out from the other natural products in the current investigations, as it is the only molecule for which the configurational discrimination via the *Q*-factors failed for HF and was significantly reduced in functionals with a larger exact exchange admixture.

To further elucidate which property of the shielding tensor causes this reduced discrimination, artificial shielding tensors have been constructed by using (i) the eigenvalues of each DFA, combined with the eigenvectors of KT2 and (ii) the eigenvectors of each DFA together with the eigenvalues of KT2. KT2 was chosen based on its pronounced discrimination of the two configurations for weizhouochrone A. For a selection of DFAs from GH, RSH, LH, and RSLH as well as Hartree–Fock, the *Q*-factors from the artificial CST are gathered in Table 2.

**Table 2:** *Q*-Factors for Weizhouochrone A with the Artificially Constructed Shielding Tensors Using KT2 as Reference

	*Q*-factor		
	original	Eigvec(KT2)	Eigval(KT2)
KT2	0.047	0.047	0.047
HF	0.093	0.096	0.047
B3LYP	0.064	0.062	0.048
ωB97M-V	0.072	0.070	0.049
scLH21ct-SVWN-m	0.059	0.055	0.050
ωLH22t	0.077	0.071	0.050

Interestingly, when comparing both scenarios for these artificially constructed CSTs, it is noticeable that only the use of KT2 eigenvalues in connection with the eigenvectors of the original tensors [scenario (ii)] shows a significant impact and provides data very close to the KT2 results, whereas for scenario (i), the overall *Q*-factors are essentially unchanged in comparison to the original (unaltered, hence DFA specific) CSTs (similar results are also observed for the other DFAs; see Table S7 in Supporting Information).

Similar to the discussion of the data from the NS372 test set, we could see here that for these natural compounds, the direction criterion is reproduced to an equal quality, while the shape criterion is more sensitive to the chosen DFAs and influences the resulting *Q*-factors more significantly.

#### 4.2.2 Conformational Ensembles

**Figure 5:**
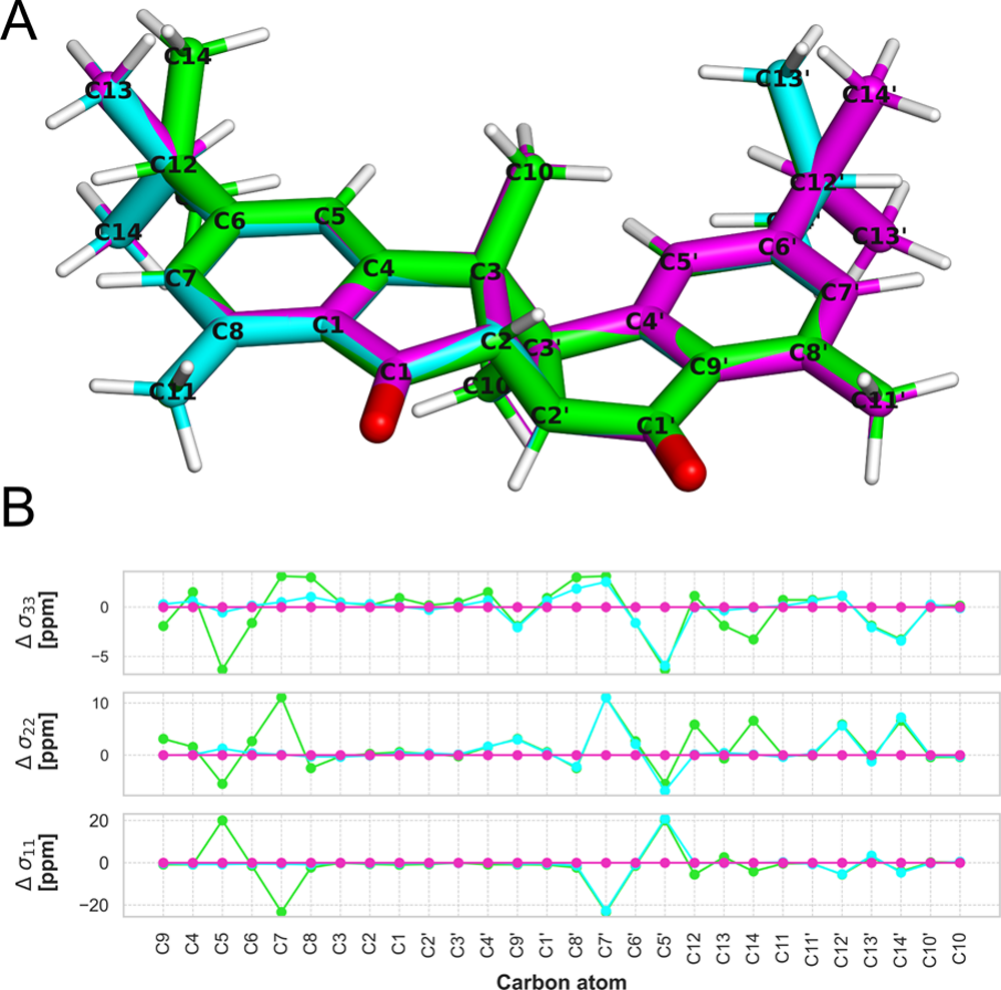
(A) Overlay of CONF1 (green), CONF2 (blue), and CONF3 (magenta) of weizhouochrone A. (B) Differences between the eigenvalues of the calculated symmetrized chemical shielding tensors using B3LYP/pcSseg-2 for the different conformers marked by the colors according to the structures. CONF3 has been used as a reference.

So far, we have not discussed the structural models we used for the RCSA analysis, as this was already systematically studied in their respective publications.^6,9^ As pointed out in these publications, a conformation ensemble is necessary to properly describe the conformational space of more flexible molecules such as **5** and **6**. However, one detail that has not been thoroughly addressed is whether it is necessary to calculate the shielding tensor for each conformer individually. The assumption is that the CST of the same nucleus in different conformers only differs in their directionality (i.e., their eigenvectors). If this is true, one could in principle save computational cost by calculating the CST for one conformer and then rotating each tensor to fit the other conformers. This, however, seems not to be the case, as can be seen, for example, in Figure 5 for the different conformers that were found for weizhouochrone A. The three conformers show mostly the same core structure and only differ in the conformation around the C12 and C12′ carbon atoms. CONF1 differs from CONF2 and CONF3 by a rotation around the C6–C12 bond and CONF2 differs from CONF1 and CONF3 by a similar rotation around the C6′–C12′ bond. When comparing CONF1 to the other two conformers, larger differences in the PC σ_33_ of C5 and C7 are observed, challenging the assumption of rotatability. The same is true for C5′ and C7′ when comparing CONF2 to CONF1 and CONF3. This alludes to the fact that the chemical shielding tensors for different conformers differ not only in the orientation but in fact also in their shape. In summary, it is useful to calculate the CST for every conformer individually, as an approximation by simple rotation may introduce a non-negligible error.

## 5 Conclusions

In this study, we have performed an extensive benchmark of ^13^C shielding anisotropies of various DFAs (and Hartree–Fock) against the CCSD(T) reference data. Similar to the isotropic shielding data, well performing functionals are among mGGAs and LHs, which especially show great potential in their strong-correlation generalization, as exemplified especially by the outstanding performance of the scLH21ct-SVWN-m functional. Differences are observed in the ordering of the DFAs: besides TPSS-based functionals, we find some more recent GGA models to perform accurately, with the lowest maximum errors in the diverse carbon data set. Some further LHs and mGGAs perform particularly well, which are not among the top performing functionals for the isotropic shielding data. However, highly parametrized functionals also give large deviations for the shielding anisotropies as observed for isotropic shielding data sets.

Furthermore, we have shown that the shape and direction criteria provide additional information about the computed shielding tensors. This extends the isotropic and anisotropic shielding data toolkit. For nuclei such as carbon, the eigenvectors are very well-defined in comparison to the CCSD(T) reference data, yet the shape criterion, defined as the deviation of the PCs vector or similarly as the deviation of the full unsymmetrized shielding tensor from the reference data, correlates only loosely with the isotropic shielding data and was shown to be a key factor in the description of RCSAs. Interestingly, in the analysis of RCSAs, the previously mostly employed B3LYP functional does not provide particularly good results in either of these test-set data. As it additionally comes at a similar cost as better performing LHs and is more computationally demanding than the semilocal DFAs, we suggest employing either semilocal models or more accurate exact-exchange dependent functionals.

Next, we evaluated the impact of different functionals on the analysis of RCSAs for the stereochemical elucidation of natural products. We showed that the discrimination of the configurations is relatively insensitive to the choice of DFA, although this depends on the system. Finally, our comparative study suggests that it is more beneficial for the RCSA to calculate the shielding tensors for all conformers individually rather than relying on the assumption that the chemical shielding tensors can be transferred within a conformational ensemble.

## Data Availability

Data set deposited on Zenodo contains: (1) complete shielding tensors for carbon nuclei in the NS372 test set, (2) coordinate files of the molecules in the NS372 used in this study, (3) complete shielding tensors for the natural products, and (4) input and output files from Turbomole and ConArch^+^ calculations for the natural products. The data set can be accessed via DOI: 10.5281/zenodo.13951994.

## Supporting Information Available

Statistical data for the Maryland defined anisotropies; detailed discussion of the Haeberlen and Turbomole defined anisotropies; cosine similarities between the RDC- and RCSA-derived alignment tensors; chemical structures of the natural products used; influence of the basis set on RCSA analysis; statistical correlation of the Maryland defined anisotropies; cosine similarities for the carbon shielding tensors of the NS372 test set; cross-correlation plot of the cosine similarities with the isotropic shielding data and cross-correlation plot of the cosine similarity of the full shielding tensor and the PCs; cross-correlation between the MAE of anisotropic and isotropic shielding data; *Q*-factors for the natural products using all tested methods; *Q*-factors for all methods using the artificially constructed shielding tensors; and computational timings for the calculation of shielding tensors in Turbomole (PDF)
