# Supplementary material for: Benchmark of Density Functional Theory in the Prediction of 13C Chemical Shielding Anisotropies for Anisotropic Nuclear Magnetic Resonance-Based Structural Elucidation
Source: J Chem Theory Comput. 2025 Jan 6;21(2):871–85. doi: 10.1021/acs.jctc.4c01407 (PMC11780741; doi:10.1021/acs.jctc.4c01407)
Supplement: Supplementary file 1 — ct4c01407_si_001.pdf [file ct4c01407_si_001.pdf]

Benchmark of density functional theory in the prediction of ^{13}C chemical shielding anisotropies for anisotropic NMR based structural elucidation

Anton Florian Ketzel,[†,‡] Xiaolu Li,[‡] Martin Kaupp,[†] Han Sun,[*,†,‡] and Caspar Jonas Schattenberg[*,‡]

†*Technische Universität Berlin, Institut für Chemie, Theoretische Chemie/Quantenchemie, Sekr. C7, Straße des 17. Juni 135, D-10623, Berlin, Germany*

‡*Leibniz-Forschungsinstitut für molekulare Pharmakologie, Department for Biological Chemistry, Campus Berlin-Buch, Robert-Roessle-Str. 10, 13125 Berlin, Germany*

E-mail: hsun@fmp-berlin.de; schattenberg@fmp-berlin.de

Contents

S1 Statistical Data of the Maryland Defined Anisotropies and Functionals Ranked 1 to 10 According to MAE and max. AE. S2

S2 Haeberlen and Turbomole Defined Anisotropies S3

S3 Influence of Different Basis Sets on *Q*-Factors for RCSA S5

S4 Cosine Similarity Between RDC and RCSA Derived Alignment Tensors S6

S5 Chemical Structures of the Configurations of the Natural Products S7

S6 Statistical Correlation of Maryland Defined Anisotropy S8

S7 Cosine Similarities S9

S8 Cross-Correlations Between Averaged Cosine Similarities of the Principle-Component Vectors, Unsymmetrized Tensors and Isotropic Shielding MAE S10

S9 Cross-Correlations Between Mean Absolute Errors of the Isotropic and Maryland-Convention Anisotropic Carbon Shielding Data S11

S10 *Q*-Factors for a Larger Set of Functionals S12

S11 Computational Timings **S15**

S12 Q-Factors of the Artificially Constructed Chemical Shielding Tensors from Different DFAs and KT2 **S16**

S1 Statistical Data of the Maryland Defined Anisotropies and Functionals Ranked 1 to 10 According to MAE and max. AE.

Table S1: Statistical data for the analysis of the anisotropies calculated with different methods compared to CCSD(T). The Maryland ordering has been employed.

Method	MSE	MAE	SD	max. AE
Hartree-Fock	14.08	17.39	23.19	134.81
S-VWN	15.84	16.24	18.13	135.74
B97-D	4.07	6.17	8.27	32.32
BLYP	9.91	10.38	11.03	49.85
BP86	8.40	9.07	9.88	43.60
HCTH	4.40	6.19	7.76	28.52
KT1	-1.80	7.13	10.46	49.94
KT2	-0.89	6.78	10.07	45.60
KT3	-2.22	6.94	10.39	47.36
PBE	9.00	9.64	10.56	57.02
M06-L	4.04	7.10	11.05	56.78
B97M-V	-2.37	5.67	8.58	39.54
MN15-L	-8.27	9.24	11.23	58.14
rSCAN	6.17	7.00	7.73	34.21
τHCTH	1.97	5.98	8.69	28.05
TPSS	3.93	5.49	7.55	34.30
VSXC	1.41	5.76	8.90	49.67
B3LYP	12.48	12.55	12.21	53.69
B97-2	7.91	8.16	8.66	38.63
BHLYP	14.76	14.93	16.70	91.88
M06	18.63	18.66	18.30	73.09
M06-2X	21.91	22.18	25.86	159.22
MN15	15.70	15.93	18.04	101.97
mPW1PW	11.77	11.83	12.22	56.72
PBE0	11.71	11.77	12.22	57.28
PW6B95	12.91	13.03	13.59	61.77
TPSSh	5.44	6.03	7.38	36.75
CAM-B3LYP	16.07	16.17	16.56	87.44
ωB97M-V	11.73	11.90	13.03	74.04
LH07s-SVWN	9.90	10.10	10.13	45.50
LH07t-SVWN	7.80	8.05	8.52	39.76
LH12ct-SsifPW92	6.48	6.86	8.20	45.25
LH12ct-SsirPW92	6.62	6.95	7.98	42.48
LH14t-calPBE	7.79	7.98	8.22	37.28
LH20t	7.34	7.59	8.57	44.09
LHJ14	13.32	13.46	13.03	58.97
mPSTS-noa2	3.72	5.24	7.18	33.46
ωLH22t	11.23	11.45	12.95	74.26
scLH21ct-SVWN-m	3.44	4.27	5.26	23.23
scLH22t	7.15	7.40	8.22	41.58
scLH22ta	6.25	6.82	7.42	39.06
scLH23t-mBR	7.03	7.28	8.20	42.85

Table S2: Functionals ranked 1 to 10 according to MAE (left) and max. AE (right).

rank	functional	MSE	SD	MAE	max. AE
1	scLH21ct-SVWN-m	3.06	5.06	3.97	23.23
2	mPSTS-noa2	3.22	6.83	4.83	33.46
3	TPSS	3.48	7.23	5.06	34.30
4	TPSSh	4.87	7.01	5.54	36.75
5	B97M-V	-2.43	9.20	5.60	58.68
6	VSXC	1.09	9.21	5.63	49.67
7	τHCTH	1.66	8.74	5.72	39.05
8	B97-D	3.66	8.20	5.89	32.32
9	HCTH	3.97	7.76	5.96	28.52
10	LH12ct-SsifPW92	5.77	7.60	6.20	45.25

rank	functional	MSE	SD	MAE	max. AE
1	scLH21ct-SVWN-m	3.06	5.06	3.97	23.23
2	HCTH	3.97	7.76	5.96	28.52
3	B97-D	3.66	8.20	5.89	32.32
4	mPSTS-noa2	3.22	6.83	4.83	33.46
5	rSCAN	5.56	7.70	6.56	34.21
6	TPSS	3.48	7.23	5.06	34.30
7	TPSSh	4.87	7.01	5.54	36.75
8	LH14t-calPBE	7.07	7.69	7.28	37.28
9	B97-2	7.19	8.27	7.55	38.63
10	τHCTH	1.66	8.74	5.72	39.05

S2 Haeberlen and Turbomole Defined Anisotropies

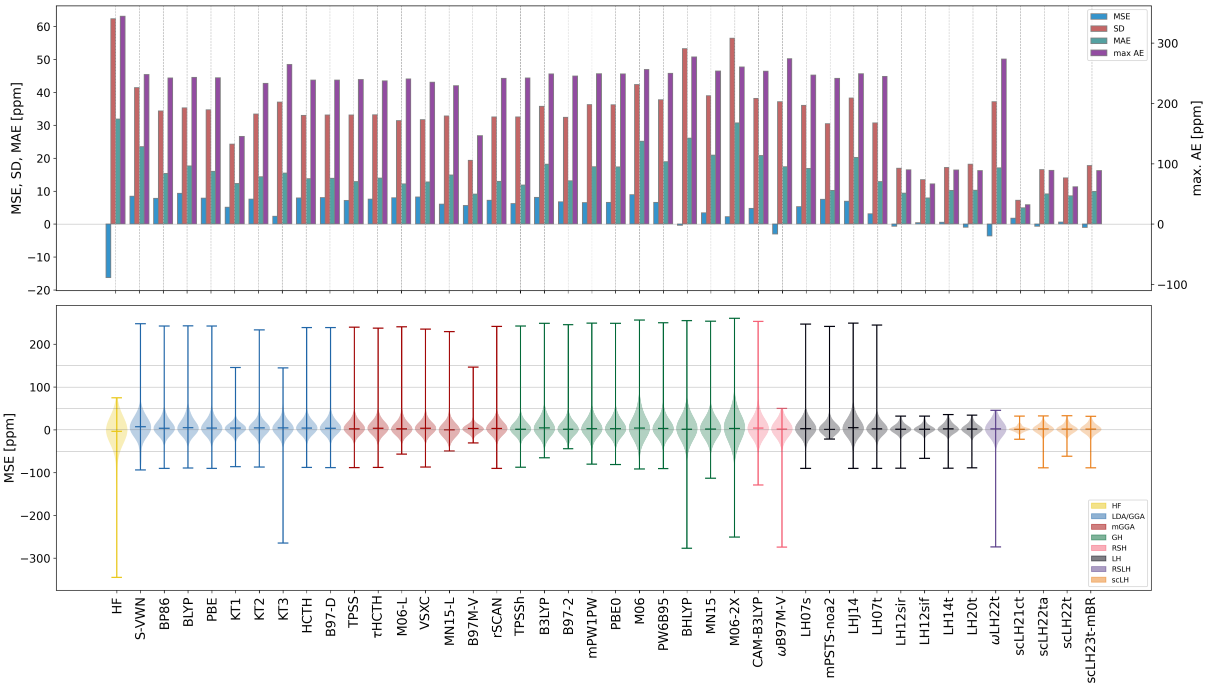

Figure S1: (Top) Statistical data, (bottom) violin plots of the mean signed errors with respect to CCSD(T) reference data of shielding anisotropies following the Haeberlen convention according to eqs. 5 and 6

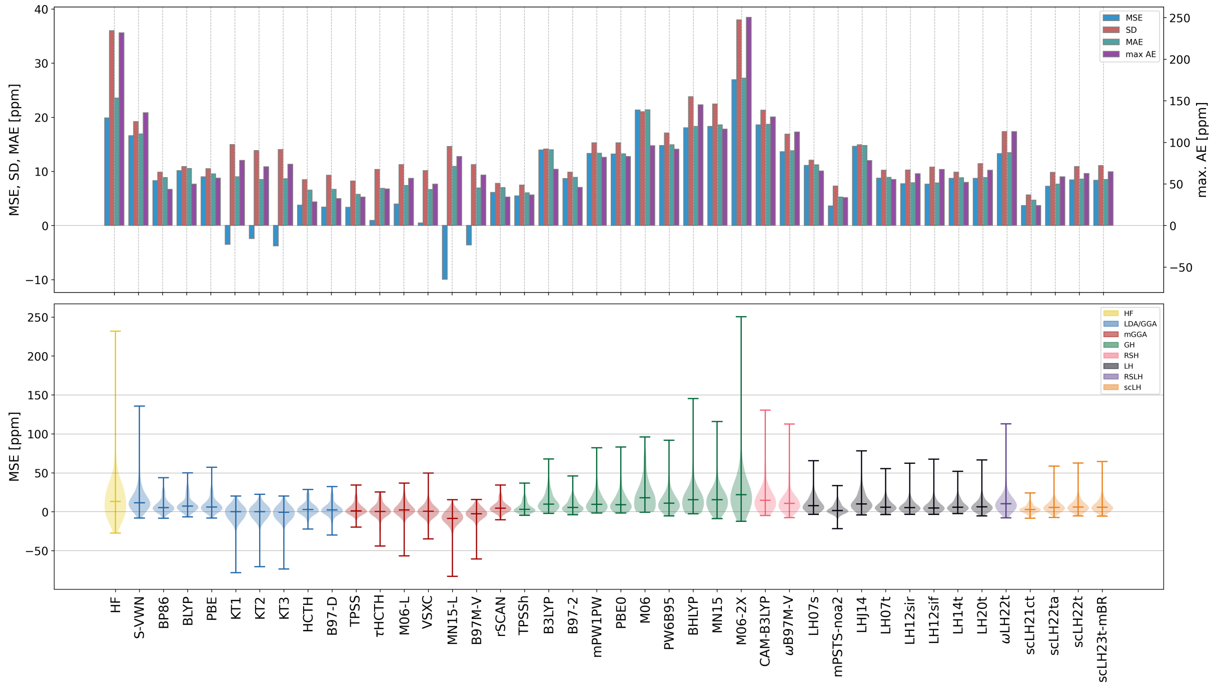

Figure S2: (Top) Statistical data, (bottom) violin plots of the mean signed errors with respect to CCSD(T) reference data of shielding anisotropies following the Turbomole definition according to eq. 8

The Haeberlen ordering of the principle components (Figure S1) is, as mentioned in the section 2 of the main text prone to sign changes upon subtle differences of the principle components as computed with different DFAs and methods. This reordering gives partly extreme deviations of the individual data points and statistical data for the majority of DFAs. Interestingly, only a few LH and scLH functionals employing the t-LMF and either calibration or larger exact exchange admixture are robust against these sign changes and hence give a similar representation of the principal components as the CCSD(T) reference data. As a consequence for these functionals the statistical data is comparable to the other two statistics, whereas in all other cases, deviations are much larger.

The Turbomole definition of the anisotropy following eq. 8 (as depicted in Figure S2) throughout gives slightly larger values than those obtained from eq. 7 and discussed in the main text. The ordering, however, and the overall performance of the functionals is not significantly affected, hence we can draw the same conclusions as from the discussion in the main text.

S3 Influence of Different Basis Sets on Q-Factors for RCSA

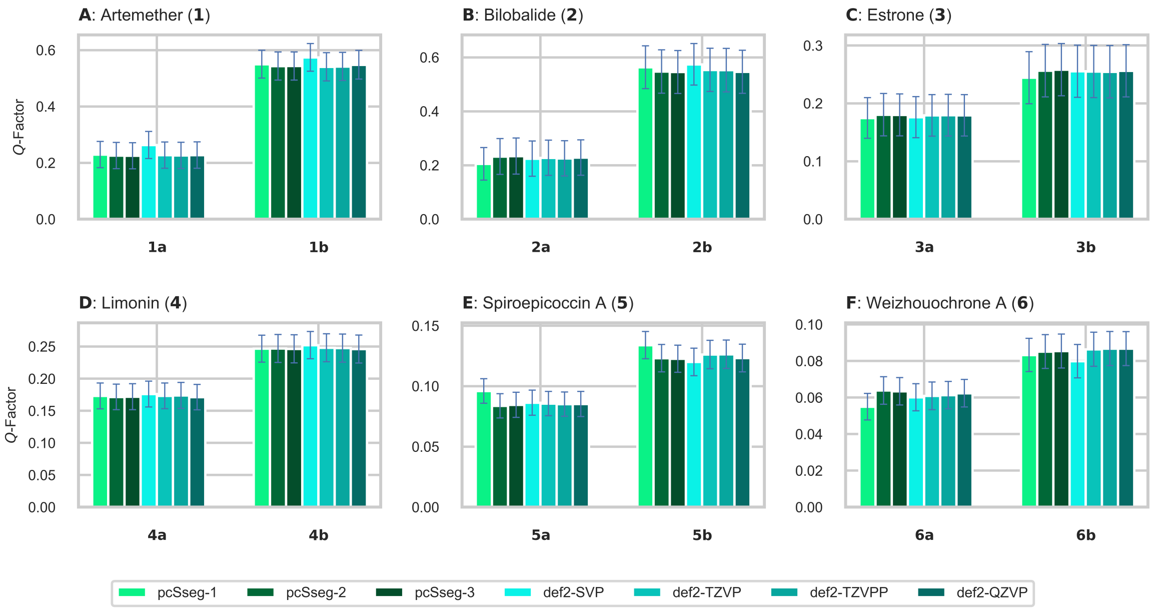

Figure S3: Q-Factors of the correct and alternative configurations of the natural products using different basis sets for the DFT calculations. All calculations have been done using B3LYP and COSMO implicit solvation for methanol. Errors are estimated by a Monte Carlo approach implemented in ConArch$^+$.

S4 Cosine Similarity Between RDC and RCSA Derived Alignment Tensors

Table S3: Cosine similarities between the RCSA- and RDC-derived alignment tensors from their respective SVD fits using CONARCH$^+$.

**Functional**	Artemether		Bilobalide		Estrone		Limonin		Spiroepicoccin A		Weizhouochrone A	
	**1a**	**1b**	**2a**	**2b**	**3a**	**3b**	**4a**	**4b**	**5a**	**5b**	**6a**	**6b**
HF	17.9	93.6	12.3	103.3	43.6	91.9	18.2	21.2	62.8	36.9	8.1	60.8
S-VWN	19.4	108.1	11.5	95.1	42.2	81.0	14.1	24.8	52.1	88.5	6.6	62.0
B97-D	17.6	109.6	10.8	95.0	41.8	84.9	14.5	25.5	59.9	86.7	7.4	62.9
BLYP	17.8	109.7	11.2	94.9	43.6	84.1	14.5	25.0	59.9	87.4	6.2	62.2
BP	18.5	108.9	11.0	95.3	41.5	84.2	14.4	25.3	59.6	86.6	7.0	62.6
HCTH	18.4	112.1	9.6	96.2	38.3	88.0	15.6	25.4	61.0	84.1	10.3	66.3
KT1	17.2	107.8	10.7	93.6	41.6	86.3	13.6	27.7	59.3	86.9	8.7	64.8
KT2	17.2	106.3	10.7	94.0	40.8	86.1	13.7	27.1	59.4	86.7	8.8	65.0
KT3	17.1	108.5	10.2	94.9	40.7	87.2	14.4	26.5	65.2	85.7	9.0	65.0
PBE	18.0	103.8	11.2	97.2	41.2	87.0	15.8	24.2	60.8	83.5	8.1	62.2
M06-L	18.2	109.3	10.8	95.5	40.4	89.3	14.7	25.7	60.2	83.6	6.5	61.6
B97M-V	17.5	101.9	11.4	97.9	43.8	87.7	15.9	23.6	67.7	81.5	9.9	62.0
MN15-L	16.7	107.5	11.0	95.2	42.2	88.0	14.6	27.5	64.6	83.7	7.6	63.6
rSCAN	18.3	107.8	11.1	95.3	41.0	85.0	14.7	25.3	59.6	87.0	7.1	63.2
τHCTH	18.4	112.1	9.6	96.2	38.3	88.0	15.6	25.4	61.0	84.1	10.3	66.3
TPSS	17.0	107.6	11.1	95.4	41.2	84.5	15.2	24.9	59.9	85.9	6.8	62.5
VSXC	13.6	104.3	11.7	94.0	44.9	84.3	14.5	28.1	59.5	88.2	8.7	61.1
B3LYP	17.3	104.4	11.4	96.9	43.2	86.2	15.4	24.1	65.4	84.3	7.4	61.8
B3LYP def2-SVP	15.9	105.9	11.6	96.3	43.6	84.3	16.3	24.3	61.1	83.4	6.6	61.3
B3LYP def2-TZVP	17.0	105.2	11.3	96.4	42.9	86.3	15.6	24.4	60.6	86.0	7.3	62.0
B3LYP def2-TZVPP	16.8	105.2	11.3	96.4	42.6	86.5	15.6	24.2	60.7	86.1	7.1	61.9
B3LYP def2-QZVP	17.1	104.4	11.2	96.8	42.4	86.0	15.5	24.1	65.2	84.2	7.0	61.9
B3LYP pcSseg-1	15.9	102.5	10.7	96.1	39.4	89.3	15.8	25.1	60.8	83.5	9.1	63.2
B3LYP pcSseg-3	17.3	104.4	11.4	96.9	43.2	86.2	15.4	24.1	65.4	84.3	7.4	61.8
B97-2	17.5	104.8	11.1	97.0	41.0	87.2	15.7	24.4	60.8	83.5	8.3	62.5
BHLYP	17.1	98.4	11.7	99.6	42.9	88.5	16.7	22.8	65.9	82.4	8.1	61.3
M06	18.7	103.8	10.4	96.6	41.6	90.7	15.3	24.5	61.3	82.9	6.6	61.6
M06-2X	17.5	99.0	11.9	99.7	43.0	86.6	17.5	22.3	65.8	82.7	7.6	61.0
MN15	16.7	107.5	11.0	95.2	42.2	88.0	14.6	27.5	64.6	83.7	7.6	63.6
mPW1PW	17.8	103.4	11.3	97.3	41.0	86.9	15.9	24.0	60.9	83.4	8.0	62.2
PBE0	18.0	103.8	11.2	97.2	41.2	87.0	15.8	24.2	60.8	83.5	8.1	62.2
PW6B95	17.5	102.0	11.4	97.5	42.1	86.2	16.2	23.8	61.0	83.6	7.7	61.6
TPSSh	16.9	105.4	11.3	96.4	41.1	85.6	15.7	24.5	60.4	83.9	7.3	62.3
CAM-B3LYP	17.2	102.0	11.8	98.2	43.8	86.0	15.9	23.1	66.2	82.9	8.3	61.4
ωB97M-V	17.5	101.9	11.4	97.9	43.8	87.7	15.9	23.6	67.7	81.5	9.9	62.0
LH07s-SVWN	16.3	105.6	11.3	97.1	40.7	85.1	15.8	23.8	60.6	84.0	8.3	62.3
LH07t-SVWN	14.9	104.5	11.9	97.4	41.5	82.4	16.1	23.7	60.0	86.1	8.5	62.3
LH12ct-SsifPW92	14.2	103.8	12.1	98.0	40.8	82.9	16.5	23.6	65.0	84.3	9.7	62.8
LH12ct-SsirPW92	14.3	103.9	12.0	97.9	41.0	82.8	16.4	23.6	65.0	84.4	9.3	62.6
LH14t-calPBE	14.8	103.7	11.9	97.4	41.9	82.9	16.0	23.8	64.8	86.1	8.8	62.3
LH20t	13.8	101.1	12.3	98.5	42.2	82.4	16.8	23.3	65.1	84.3	9.5	62.0
LHJ14	17.0	103.6	11.4	96.3	42.8	83.7	15.1	24.0	60.6	84.0	7.5	61.6
mPSTS-noa2	16.1	104.7	11.2	96.6	40.2	86.3	15.9	24.6	60.5	83.3	8.0	62.4
ωLH22t	15.1	101.3	12.1	98.8	43.2	82.9	16.6	22.9	66.1	82.9	10.1	61.7
scLH21ct-SVWN-m	14.5	106.0	12.0	97.0	42.2	80.3	15.6	23.0	65.1	84.2	7.6	62.5
scLH22t	14.2	101.2	12.3	98.4	42.4	82.1	16.6	23.1	65.0	84.4	9.1	62.1
scLH22ta	14.0	103.0	12.4	97.7	43.2	78.8	16.1	22.4	65.1	84.5	8.4	62.1
scLH23t-mBR	13.9	101.1	12.3	98.5	42.2	82.4	16.8	23.4	65.0	84.3	9.5	62.0

S5 Chemical Structures of the Configurations of the Natural Products

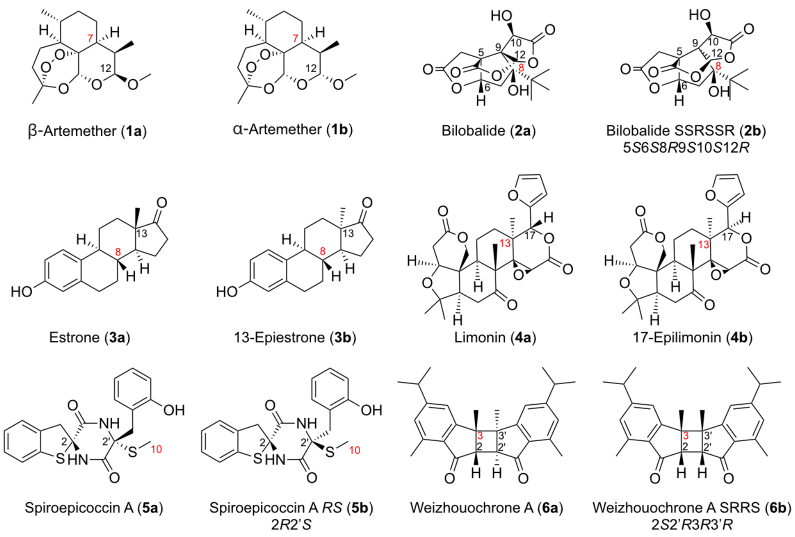

Figure S4: Chemical structures of all natural products and the studied alternative configurations. The stereocenters are marked with black numbers and the reference atoms for the RCSA analysis with red numbers. For Bilobalide and Weizhouochrone A the reference atom is a stereocenter as well.

S6 Statistical Correlation of Maryland Defined Anisotropy

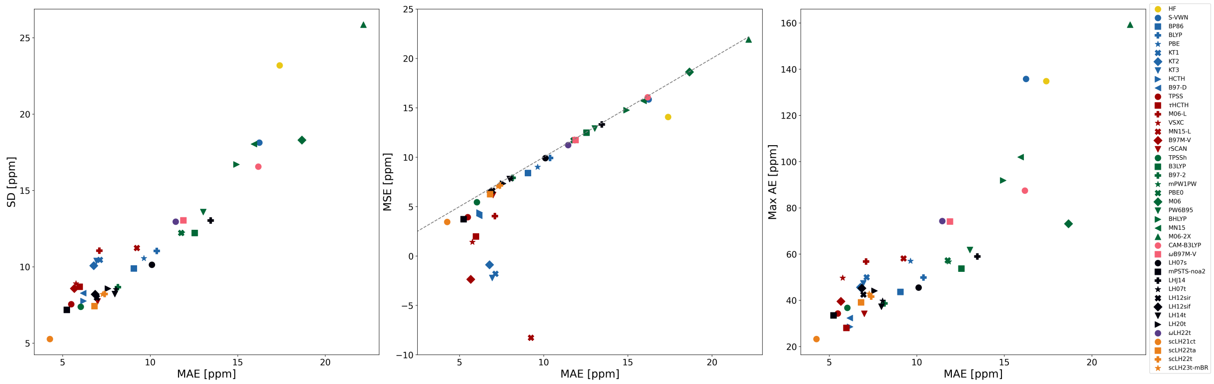

Figure S5: MAE plotted against SD, MSE and max. AE as a visualization of functionals performances. The diagonal line in the MSE vs. MAE plot reflects MSE=MAE, i.e. systematic overestimation

S7 Cosine Similarities

Table S4: (first three panels) Ordered deviations of the cosine similarities of methods and DFAs against the CCSD(T) reference data; (last panel) Average of the cosine similarities, calculated as $N^{-1} * \sum_i^N x_i$, where x_i is the ith cosine similarity of the test set.

Eigenvector	X					Y					Z					MAD		
Deviation[a]	≤ 1	≤ 2	≤ 4	≤ 10	≤ 20	≤ 1	≤ 2	≤ 4	≤ 10	≤ 20	≤ 1	≤ 2	≤ 4	≤ 10	≤ 20	X	Y	Z
Hartree-Fock	37	6	8	10	2	32	8	9	11	2	83	5	1	1	0	1.721	1.926	0.251
S-VWN	48	9	2	3	1	46	7	3	4	2	86	1	1	1	1	0.878	1.300	0.396
BP86	44	13	2	4	0	41	12	3	5	1	86	1	1	1	1	0.904	1.272	0.373
BLYP	40	13	7	3	0	36	13	8	5	0	82	4	2	2	0	1.058	1.413	0.384
PBE	43	13	3	4	0	40	13	3	5	1	86	1	1	1	1	0.952	1.315	0.381
KT1	41	8	10	4	0	38	8	10	6	0	85	2	1	2	0	1.133	1.377	0.327
KT2	40	14	6	3	0	39	12	6	5	0	85	2	1	2	0	1.036	1.283	0.315
KT3	41	10	9	3	0	38	9	9	6	0	81	6	0	3	0	1.081	1.376	0.370
HCTH	43	11	4	5	0	39	10	5	8	0	82	2	3	3	0	1.048	1.424	0.423
B97-D	43	11	5	4	0	40	10	6	6	0	85	1	2	2	0	0.945	1.277	0.370
TPSS	42	14	4	3	0	39	14	4	4	1	83	4	1	1	1	0.859	1.209	0.357
τHCTH	45	10	4	4	0	43	8	5	5	1	84	2	2	1	1	0.879	1.218	0.377
M06-L	50	6	4	3	0	46	6	5	4	1	82	4	2	1	1	0.825	1.140	0.353
VSXC	50	8	2	3	0	46	7	6	3	0	85	1	3	1	0	0.731	0.879	0.252
MN15-L	50	8	2	3	0	49	6	3	4	0	84	3	1	2	0	0.667	0.780	0.245
B97M-V	41	15	4	3	0	39	13	6	4	0	82	4	3	1	0	0.844	1.028	0.310
rSCAN	51	5	4	3	0	48	5	4	5	0	86	1	1	2	0	0.643	0.850	0.259
TPSSh	53	5	4	1	0	48	6	5	3	0	85	2	1	2	0	0.548	0.837	0.284
B3LYP	57	1	5	0	0	52	2	6	2	0	85	2	1	2	0	0.418	0.654	0.234
B97-2	57	1	5	0	0	53	1	6	2	0	85	2	1	2	0	0.385	0.606	0.220
mPW1PW	56	3	4	0	0	51	5	5	1	0	86	2	1	1	0	0.425	0.647	0.206
PBE0	56	3	4	0	0	53	2	6	1	0	86	1	2	1	0	0.417	0.629	0.202
M06	48	10	3	2	0	44	11	3	4	0	86	2	0	2	0	0.686	0.857	0.191
PW6B95	54	6	3	0	0	50	6	4	2	0	86	1	1	2	0	0.405	0.614	0.203
BHLYP	43	12	6	2	0	41	11	8	2	0	88	1	1	0	0	0.748	0.820	0.121
MN15	45	9	7	2	0	42	8	9	3	0	87	1	1	1	0	0.785	0.948	0.188
M06-2X	41	14	5	3	0	37	16	6	3	0	84	5	1	0	0	0.897	0.994	0.151
CAM-B3LYP	54	7	2	0	0	48	11	3	0	0	85	4	1	0	0	0.425	0.539	0.136
ωB97M-V	55	7	1	0	0	51	8	3	0	0	87	1	2	0	0	0.359	0.461	0.126
LH07s	56	2	4	1	0	52	1	6	3	0	86	0	2	2	0	0.402	0.624	0.206
mPSTS-noa2	56	3	3	1	0	51	3	5	3	0	85	1	2	2	0	0.466	0.765	0.286
LHJ14	56	3	2	2	0	51	4	3	4	0	86	1	1	2	0	0.438	0.653	0.191
LH07t	56	4	2	1	0	52	5	3	2	0	85	3	1	1	0	0.415	0.613	0.181
LH12sir	56	5	1	1	0	51	7	3	1	0	84	5	1	0	0	0.349	0.519	0.165
LH12sif	57	4	1	1	0	53	6	2	1	0	85	4	1	0	0	0.347	0.504	0.163
LH14t	57	3	2	1	0	53	4	3	2	0	85	3	1	1	0	0.417	0.614	0.178
LH20t	58	3	1	1	0	53	5	2	2	0	85	3	1	1	0	0.375	0.524	0.171
ωLH22t	56	5	1	1	0	52	7	2	1	0	87	2	1	0	0	0.403	0.486	0.119
scLH21ct	52	7	3	1	0	48	7	5	2	0	85	2	2	1	0	0.590	0.771	0.182
scLH22ta	51	10	0	2	0	47	11	1	3	0	85	3	1	1	0	0.547	0.735	0.187
scLH22t	59	2	1	1	0	54	4	2	2	0	85	3	1	1	0	0.376	0.534	0.175
scLH23t-mBR	59	2	1	1	0	54	4	2	2	0	85	3	1	1	0	0.393	0.545	0.173

[a] Values are assigned just once, i.e. categories are sorted ascending, so the categories become $0 \leq x \leq 1$, $1 < x \leq 2$, $2 < x \leq 4$, $4 < x \leq 10$ and $10 < x \leq 20$.

S8 Cross-Correlations Between Averaged Cosine Similarities of the Principle-Component Vectors, Unsymmetrized Tensors and Isotropic Shielding MAE

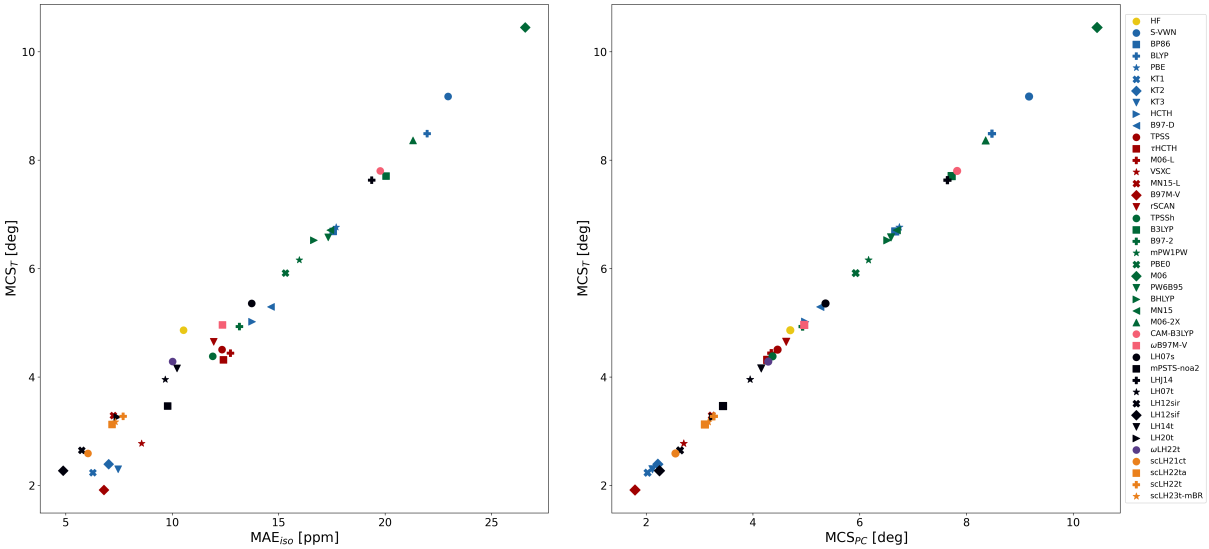

Figure S6: (Left) MAE of isotropic carbon shielding data plotted against mean value of cosine similarities of unsymmetrized DFA against CCSD(T) tensors; (Right) mean value of cosine similarities of unsymmetrized DFA against CCSD(T) tensors plotted against mean value of cosine similarities of DFAs against CCSD(T) principle component vectors $((\sigma_{11}, \sigma_{22}, \sigma_{33}))$.

S9 Cross-Correlations Between Mean Absolute Errors of the Isotropic and Maryland-Convention Anisotropic Carbon Shielding Data

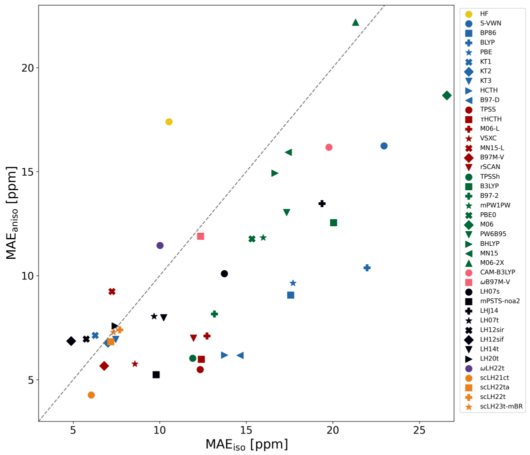

Figure S7: Cross correlation of anisotropic (calculated using Equation 7) vs. isotropic carbon shielding mean absolute errors against CCSD(T) reference data.

S10 Q-Factors for a Larger Set of Functionals

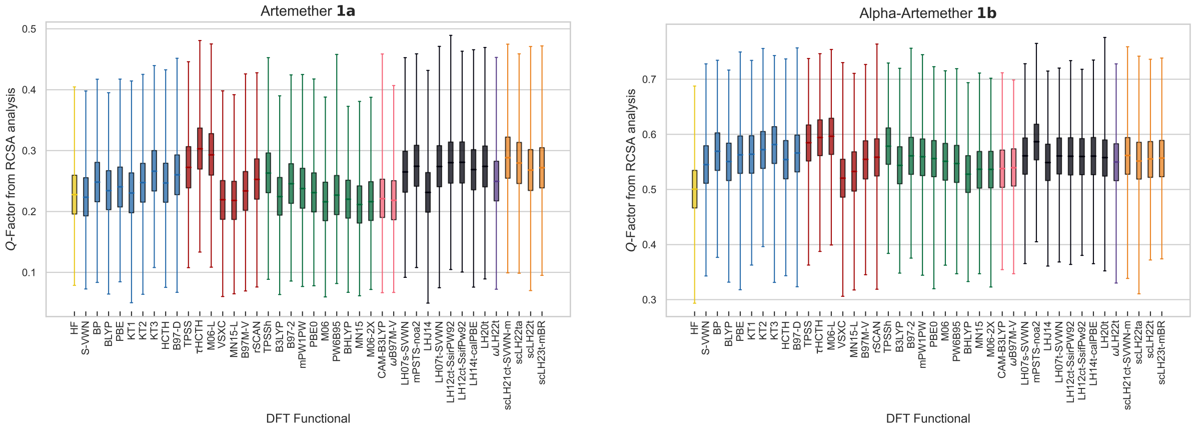

Figure S8: Q-Factors with different functionals and HF for (Left) β-Artemether (**1a**) and (Right) α-Artemether (**1b**). The error bars indicate the maximum and minimum Q-Factor determined from the Monte Carlo approach. Half of the Q-Factors determined through the Monte Carlo approach are inside the boxes, and the middle line represents the median.

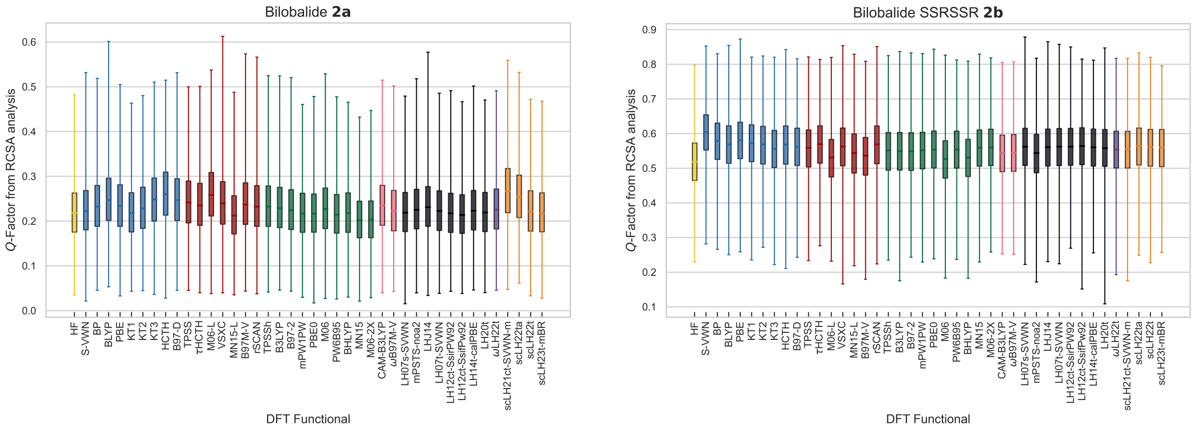

Figure S9: Q-Factors with different functionals and HF for (Left) Bilobalide (**2a**) and (Right) Bilobalide *SSRSSR* (**2b**). The error bars indicate the maximum and minimum Q-Factor determined from the Monte Carlo approach. Half of the Q-Factors determined through the Monte Carlo approach are inside the boxes, and the middle line represents the median.

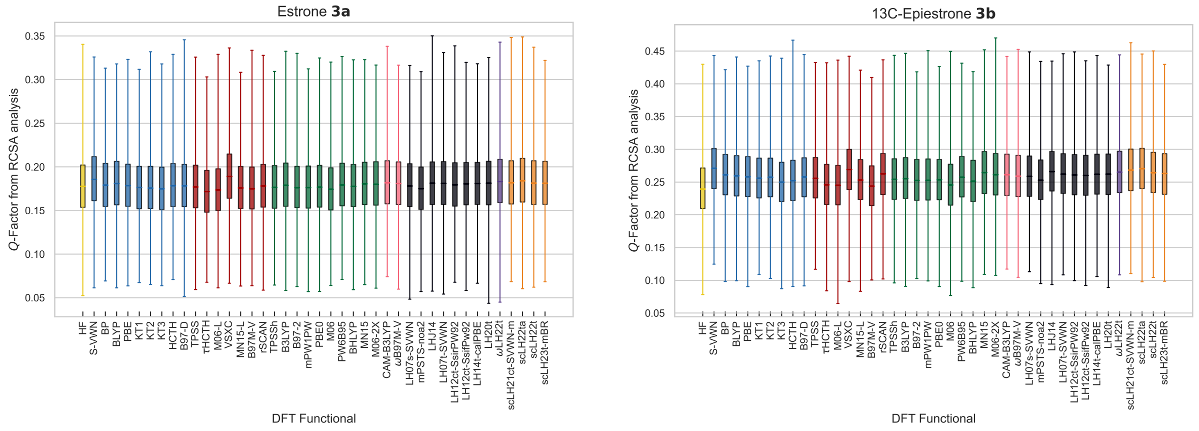

Figure S10: Q-Factors with different functionals and HF for (Left) Estrone (**3a**) and (Right) 13C-Epiestrone (**3b**). The error bars indicate the maximum and minimum Q-Factor determined from the Monte Carlo approach. Half of the Q-Factors determined through the Monte Carlo approach are inside the boxes, and the middle line represents the median.

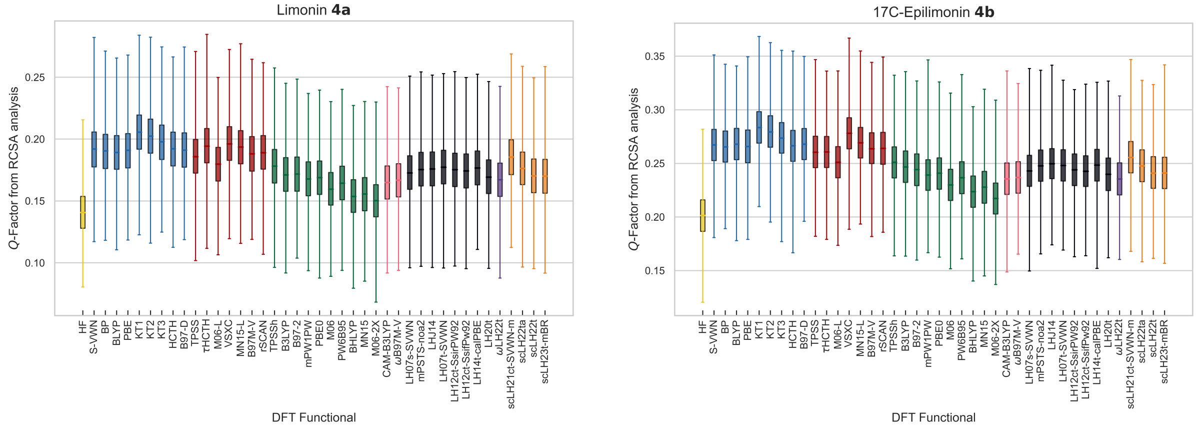

Figure S11: Q-Factors with different functionals and HF for (Left) Limonin (**4a**) and (Right) 17C-Epilimonin (**4b**). The error bars indicate the maximum and minimum Q-Factor determined from the Monte Carlo approach. Half of the Q-Factors determined through the Monte Carlo approach are inside the boxes, and the middle line represents the median.

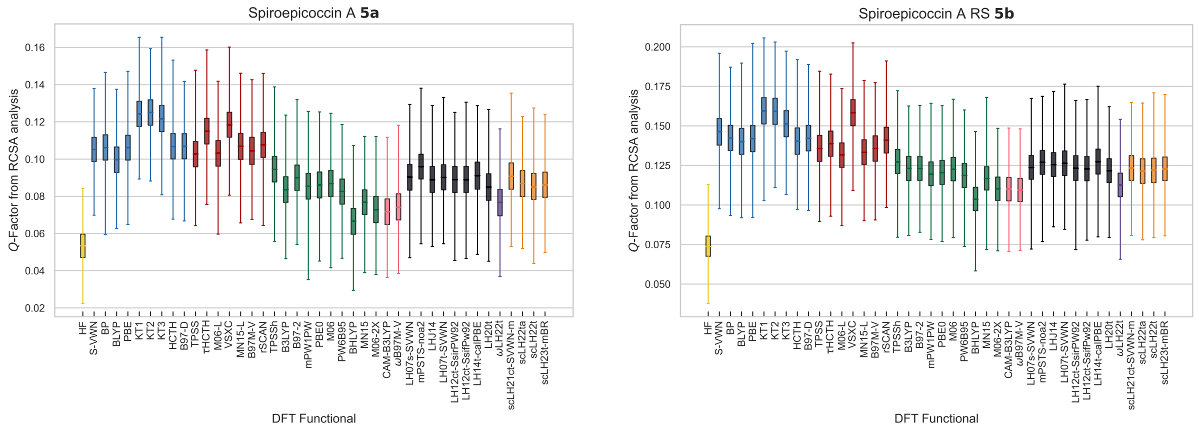

Figure S12: Q-Factors with different functionals and HF for (Left) Spiroepicoccin A (**5a**) and (Right) Spiroepicoccin A *RS* (**5b**). The error bars indicate the maximum and minimum Q-Factor determined from the Monte Carlo approach. Half of the Q-Factors determined through the Monte Carlo approach are inside the boxes, and the middle line represents the median.

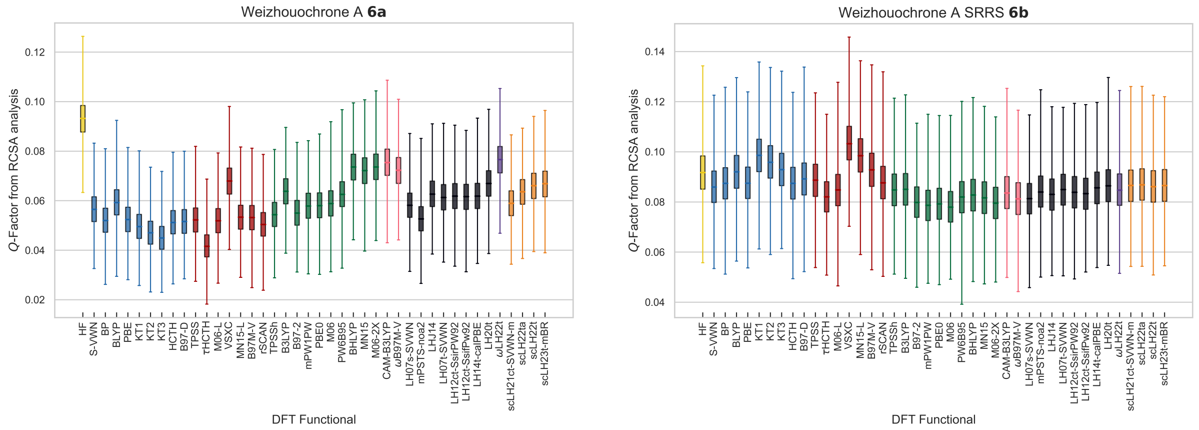

Figure S13: Q-Factors with different functionals and HF for (Left) Weizhouochrone A (**6a**) and (Right) Weizhouochrone A *SRRS* (**6b**). The error bars indicate the maximum and minimum Q-Factor determined from the Monte Carlo approach. Half of the Q-Factors determined through the Monte Carlo approach are inside the boxes, and the middle line represents the median.

S11 Computational Timings

In the following, the computational timings for a selection of DFAs for the RIDFT and MPSHIFT calculations are shown in Tab. S5 and Tab. S6. The first conformer of Weizhouochrone A (**6a**) has been used as a test structure. All calculations have been done using the same guess orbitals from the Extended Hückel Theory. The calculations have been done with the same settings as for the RCSA analysis i.e. pcSseg-2 basis set and COSMO implicit solvation. All timings were performed on a consumer Intel Core i9-13900K by selecting the same eight performance cores with the taskset command in order to minimize core variability.

Table S5: Timings for the RIDFT module have been done using the same four performance cores of an Intel i9-13900K CPU.

Method	Total Time		SCF cycles	
	CPU time [s]	Wall time [s]		Wall time per cycle [s]
KT2	604	174	18	9.7
TPSS	907	256	20	12.8
B3LYP (Analyt.)	12273	3136	17	184.5
B3LYP (Sem.Num.)[a]	15916	4003	172	235.5
ωB97M-V (Analyt.)	17348	4453	18	247.4
ωB97M-V (Sem.Num.)[a]	27734	6980	18	387.8
scLH21ct-SVWM (Sem.Num.)	17566	4416	18	245.3

[a] Using SENEX with gridsize 3.

Table S6: Timings for the MPSHIFT module have been done using the same four performance cores of an Intel i9-13900K CPU.

Method	Total Time		Separated Time		
	CPU [s]	Wall [s]	Pre-CPHF[b] [s]	CPHF cycles	CPHF cycle [s]
KT2	456	115	447	–	–
TPSS	1375	348	816	4	105
B3LYP (Analyt.)	16852	4268	4651	6	2033
B3LYP (Sem.Num.)[a]	10972	2746	2117	6	1475
ωB97M-V (Analyt.)	28623	7252	5880	9	2527
ωB97M-V (Sem.Num.)[a]	21137	5289	2832	9	2034
scLH21ct-SVWM-m (Sem.Num.)	19482	4874	8442	6	1839

[a] Using SENEX with gridsize 3.
[b] Sum of one electron, two electron, and unperturbed chemical shift contributions.

S12 Q-Factors of the Artificially Constructed Chemical Shielding Tensors from Different DFAs and KT2

Table S7: Q-Factors for Weizhouochrone A (**6a**) with the artificially constructed shielding tensors using KT2 as a reference. The Q-Factors have been calculated with the artificial shielding tensors as described before.

	Q-Factor		
	Unaltered	Eigvec(KT2)	Eigval(KT2)
HF	0.093	0.096	0.047
S-VWN	0.057	0.051	0.05
BLYP	0.059	0.056	0.049
BP	0.052	0.050	0.048
B97-D	0.052	0.050	0.048
HCTH	0.052	0.049	0.050
KT1	0.050	0.050	0.047
KT2	0.047	0.047	0.047
KT3	0.045	0.044	0.048
M06-L	0.052	0.051	0.049
PBE	0.052	0.049	0.049
rSCAN	0.051	0.049	0.048
τHCTH	0.042	0.040	0.049
M06	0.059	0.061	0.048
MN15-L	0.053	0.053	0.047
TPSS	0.052	0.050	0.048
VSXC	0.068	0.066	0.048
B3LYP	0.064	0.062	0.048
B97-2	0.055	0.055	0.048
B97M-V	0.053	0.053	0.048
BHLYP	0.074	0.074	0.047
M06-2X	0.074	0.070	0.049
MN15	0.072	0.070	0.047
mPW1PW	0.058	0.057	0.048
PBE0	0.058	0.057	0.048
PW6B95	0.063	0.061	0.048
TPSSh	0.055	0.053	0.048
CAM-B3LYP	0.075	0.073	0.048
ωB97M-V	0.073	0.070	0.049
mPSTS-noa2	0.053	0.052	0.047
LH07s-SVWN	0.058	0.055	0.049
LH07t-SVWN	0.062	0.058	0.049
LH12ct-SsifPW92	0.062	0.058	0.049
LH12ct-SsirPW92	0.062	0.058	0.049
LH14t-calPBE	0.062	0.059	0.048
LH20t	0.067	0.063	0.049
LHJ14	0.063	0.060	0.048
ωLH22t	0.077	0.072	0.049
scLH21ct-SVWN-m	0.059	0.055	0.050
scLH22t	0.066	0.062	0.049
scLH22ta	0.064	0.057	0.052
scLH23t-mBR	0.067	0.063	0.049
